# An evolutionarily conserved serine protease network mediates melanization and Toll activation in *Drosophila*

**DOI:** 10.1126/sciadv.adk2756

**Published:** 2023-12-20

**Authors:** Tisheng Shan, Yang Wang, Krishna Bhattarai, Haobo Jiang

**Affiliations:** Department of Entomology and Plant Pathology, Oklahoma State University, Stillwater, OK 74078, USA.

## Abstract

Melanization and Toll pathway activation are essential innate immune mechanisms in insects, which result in the generation of reactive compounds and antimicrobial peptides, respectively, to kill pathogens. These two processes are mediated by phenoloxidase (PO) and Spätzle (Spz) through an extracellular network of serine proteases. While some proteases have been identified in *Drosophila melanogaster* in genetic studies, the exact order of proteolytic activation events remains controversial. Here, we reconstituted the serine protease framework in *Drosophila* by biochemical methods. This system comprises 10 proteases, i.e., ModSP, cSP48, Grass, Psh, Hayan-PA, Hayan-PB, Sp7, MP1, SPE and Ser7, which form cascade pathways that recognize microbial molecular patterns and virulence factors, and generate PO1, PO2, and Spz from their precursors. Furthermore, the serpin Necrotic negatively regulates the immune response progression by inhibiting ModSP and Grass. The biochemical approach, when combined with genetic analysis, is crucial for addressing problems that long stand in this important research field.

## Teaser

A serine protease network in the fruit fly activates immune defense against pathogens.

## INTRODUCTION

Sequential activation of extracellular serine protease cascades regulates important innate immune reactions in animals, such as blood coagulation (*1*) and complement activation (*2*). Protease cascade systems also coordinate insect immune responses, notably melanization and Toll pathways, by activating prophenoloxidase (PPO) and proSpätzle (proSpz), respectively (*3*, *4*). Cascade members initially circulate in hemolymph (insect blood) as inactive zymogens and become orderly activated upon tissue damage (*5*) or pathogen invasion (*6*). To confine immune reactions and prevent excessive activation, the proteolytic process is regulated by serine protease inhibitors, especially members of the serpin superfamily (*7*). Insects have a large number of serine proteases (*8*) and serpins (*9*). However, because of the ancient divergence of their gene ancestors from those in other animal groups, the physiological roles of these encoded proteins in insects need to be determined experimentally. Over the past 25 years, genetic and biochemical approaches have led to varying degrees of success in pathway elucidation in a few insect species (*6*, *10*–*12*).

In *Drosophila melanogaster*, genetic evidence supports the existence of two cascades that link microbial infection to PPO and proSpz activation, via pathogen- and danger-associated molecular patterns. Pattern recognition receptors (PRRs) recognize bacteria by PGRP-SA (*13*) and GNBP1 (*14*) or fungi by GNBP3 (*15*) through binding to cognate polysaccharides to activate an initiating modular serine protease, ModSP (*16*), and several clip-domain serine proteases (also known as CLIPs), including Grass (*17*), Psh (*15*), Hayan (*5*, *6*), and a terminal protease Spätzle processing enzyme (SPE). SPE then converts a secreted cytokine-like molecule proSpätzle into its active form, which binds to the Toll receptor and turns on Toll signaling in fat body (*18*). In the danger signal pathway, proteases from invading pathogens are sensed directly via their cleavage of the proPsh in its bait region, leading to proSpz cleavage by SPE (*17*, *19*). The upstream cascade members that induce Toll activation also participate in melanization response (*6*). Although there is no direct evidence, it has been proposed that the PRR-Toll cascade likely diverges downstream of Psh and Hayan to activate Sp7 (*6*), which then activates PPO1 (*20*, *21*) to combat invading microbes with PO-generated reactive compounds. Note that *psh* and *Hayan* arose from a recent gene duplication and alternative splicing can produce *Hayan* transcripts similar to *psh* mRNA (*6*). It is unknown whether Hayan can sense microbial proteases and, unlike Psh, Hayan is required for wound site melanization, and its catalytic domain alone can process recombinant PPO1 to PO1 (*5*, *6*). Functional redundancy, as exemplified by the case of Psh and Hayan, leads to complexity of the serine protease cascades and limits the resolving power of genetic tools in elucidating mechanisms underlying the innate immune responses. Similarly, genetic evidence suggests the existence of another SPE-like protease capable of cleaving proSpz (*6*, *22*, *23*). Because SPE has as many as eight close paralogs (Easter, MP1, Ser7, cSP14, cSP16, cSP38, cSP61, and cSP229) (*8*), putative functional overlaps may make it difficult, if not impossible, to resolve this complicated step solely by genetic approach. Additionally, the protease acting between ModSP and Grass is unknown, leaving an important gap in our knowledge of the serine protease network (*16*). Serpins are suicide substrate inhibitors playing critical roles in the negative control of proteolytic cascades. Necrotic (Nec) was the first *Drosophila* serpin identified as a controller of the immune response (*24*). Mutations in its gene lead to spontaneous melanization and constitutive activation of the Toll pathway. While mutations in *psh* suppressed all the *nec* phenotypes (*25*) and Psh was suggested as the target of Nec (*26*), no direct interaction has been demonstrated so far (*27*). In summary, genetic studies have elegantly characterized these pathway members; however, the current impasse in the study of *Drosophila* serine protease network highlights the importance of incorporating biochemical approaches to advance our understanding of the mechanisms underlying the innate immune responses.

Biochemical studies carried out in large insects have led to detailed understanding of the proteolytic cascades. In *Tenebrio molitor*, the recognition of microbial cell wall components, including lysine-type peptidoglycan (Lys-PG) of Gram-positive bacteria or β-1,3-glucan of fungi, is accomplished by the PGRP-SA/GNBP1 complex (*28*) or GNBP3 (*29*), respectively. The recognition leads to the activation of proSpz as well as PPO through the sequential activation of three serine proteases, modular serine protease (MSP), SPE-activating enzyme (SAE), and SPE (*28*, *30*). Once activated, each protease in this cascade is regulated by a specific serpin to limit damage to the host (*31*). In *Manduca sexta*, efficient initiation of the immune protease cascades was triggered by orthologous PRRs (*32*–*34*). The difference is that PGRPs circulating in *Manduca* hemolymph bind preferentially to diaminopimelic acid-type peptidoglycan (DAP-PG) than Lys-PG (*35*, *36*). The initiation signals are subsequently amplified through a complex network of at least 12 serine proteases. The activation of Toll signaling is mediated by the sequential activation of HP14 (*32*–*34*), HP21 (*37*), HP5 (*11*), HP6 (*38*), HP8 (*39*), and Spz1. HP5 is also capable of propagating immune signaling through direct activation of Spz1 in extra-embryonic tissues (*40*). This Toll cascade branches at the position of HP21 and HP6 to activate PAP2/PAP3 (*37*, *41*) and PAP1 (*38*), respectively, which, in turn, activate a PPO heterodimer (*42*–*44*). In addition, inhibition tests indicated that a single serine protease is down-regulated by multiple serpins, whereas a single serpin can modulate the activity of several serine proteases in *M. sexta* (*45*). In conclusion, biochemical studies provide direct evidence and important insights into the molecular basis underlying the protease cascades in beetles and moths (*10*, *11*), and coupling phylogenetic relationships with biochemical characterization may break the impasse in the study of *Drosophila* serine protease network.

The presence of 52 CLIP genes in the *Drosophila* genome (*8*) has hampered a comprehensive understanding of the immune serine protease system in which CLIPs are its major components (*4*, *27*). To address this challenge, we used phylogenetic analysis and gene expression profiling to predict potential members of the immune protease pathways. Through in vitro reconstitution experiments using the purified recombinant proteins, we demonstrated that a complex network of serine proteases in the S1A subfamily may tightly regulate the defense responses in *Drosophila*, by providing a link between microbial recognition and activation of downstream immune factors. Globally, this study uncovered a complex mechanism underlying melanization and Toll activation in *Drosophila*, which is conserved among holometabolous insects and may inspire similar functional studies in other insects of practical importance.

## RESULTS

### Identification and expression of putative members of the *Drosophila* immune serine protease system

The current model of melanization and Toll pathways in *Drosophila* is still fragmentary, despite the accumulating genetic data. No direct link could be established between any of the identified serine proteases due to lack of biochemical evidence and possible presence of unidentified pathway members. As gene orthology and expression patterns are critical for inferring equivalence of function, we integrated the publicly available phylogenetic and RNA sequencing data on *Drosophila* serine proteases (fig. S1) (*8*) to identify potential counterparts of the known cascade members in *Manduca* and *Tenebrio* (*46*). Phylogenetic analysis revealed at least one ortholog of each member in *Drosophila*. All genetically characterized immune cascade members, including ModSP, Grass, Psh, Hayan, SPE, and Sp7, showed very similar patterns of expression, with high mRNA levels in most cases, and easily detected in the whole body as well as the fat body samples. cSP48 and MP1 also exhibit such expression patterns. cSP48 clusters phylogenetically with Ms-HP21 and Tm-SAE, the second proteases in their corresponding cascades. As the protease acting between ModSP and Grass is unknown (*16*), we speculate that cSP48 may play the same role as Ms-HP21 (*11*, *37*), linking ModSP (an ortholog of Ms-HP14) and Grass (an ortholog of Ms-HP5). In the case of MP1, although it formed a cluster with Dm-SPE, Ms-HP8, and Tm-SPE, and expression of the MP1 catalytic domain only led to constitutive melanization (*20*) and cleavage of Spz (*22*, *23*), *MP1* null mutants failed to show a clear role in either the melanization response or Toll pathway activity (*6*). Perhaps, MP1 only contributes to the immune responses and its redundant role needs clarification by a biochemical analysis using full-length protein. In comparison, mRNA levels of the other proteases are relatively low in whole body or are not expressed in fat body samples. Among them, Easter is the terminal protease for activating the Toll pathway that controls dorsal-ventral pattern formation during embryogenesis but is not required for Toll activation in immunity (*22*, *47*).

**Fig. 1.**
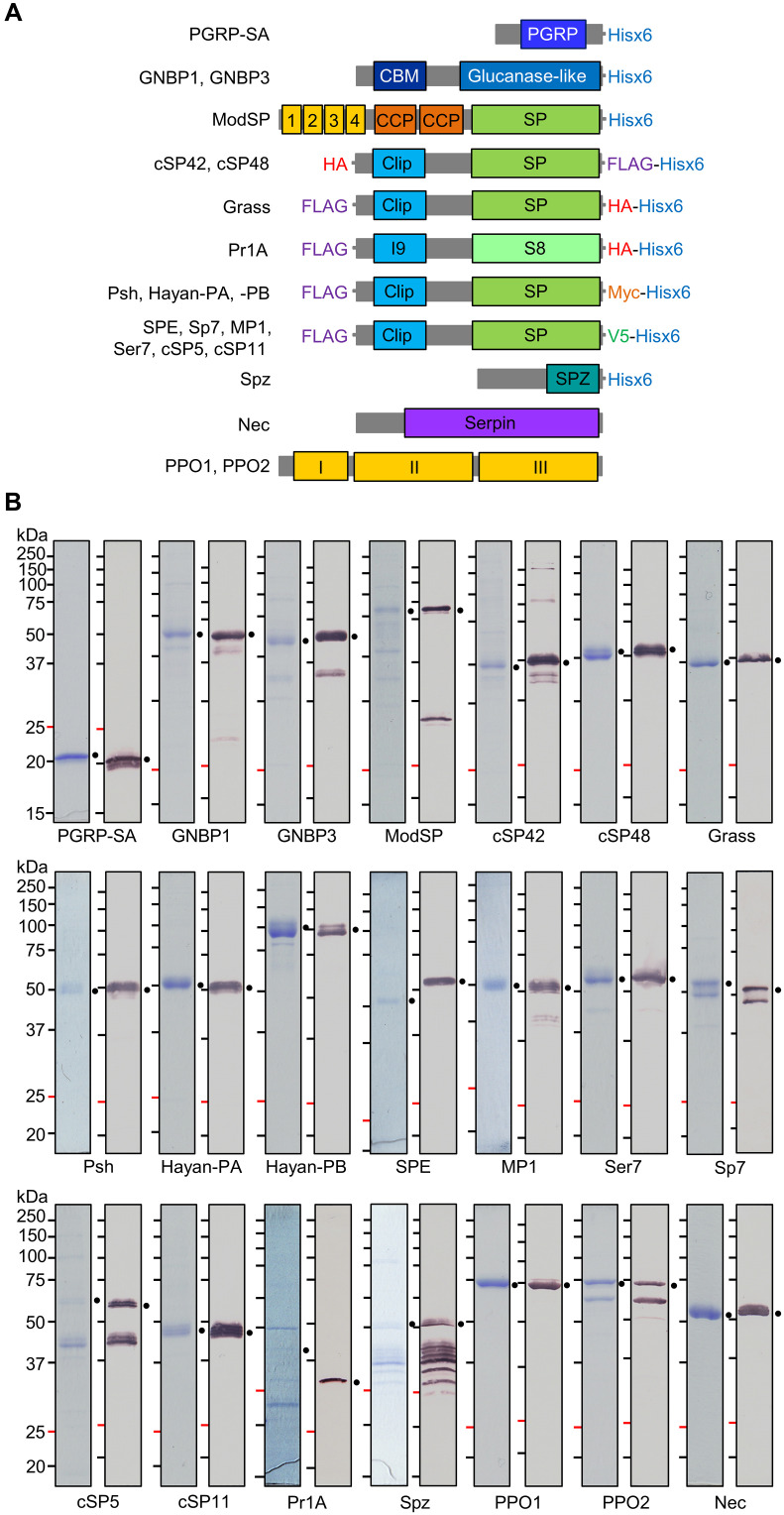
Structural features and production of the recombinant proteins. (**A**) Domain architecture and epitope tags of the recombinant proteins. The domain names are abbreviated as PGRP for peptidoglycan recognition protein domain, CBM for carbohydrate binding module, glucanase-like for β-1,3-glucanase-like domain, 1 to 4 for low-density lipoprotein receptor class A repeats 1 to 4, CCP for complement control protein or Sushi domain, SP for S1A serine protease domain, I9 for peptidase inhibitor family I9 domain, S8 for S8A serine protease domain, SPZ for cystine-knot cytokine domain in Spätzle, and I to III for PPO domains I to III. (**B**) SDS-PAGE and immunoblot analyses of the purified recombinant proteins. The proteins were treated with SDS sample buffer containing dithiothreitol (DTT) and separated by 12% (for PGRP-SA and Spz) or 10% (for the other proteins) SDS-PAGE followed by Coomassie brilliant blue staining (left) or immunoblotting (right). Sizes and positions of the *M*_r_ markers are indicated. The primary antibodies used include anti-6×His (for PGRP-SA, GNBP1, GNBP3, ModSP, cSP42, cSP48, Grass, Psh, Hayan-PA, Hayan-PB, SPE, MP1, Ser7, Sp7, cSP5, cSP11, and Spz), HA (for Pr1A), PPO1, PPO2, and Nec antibodies. Amounts of the proteins used for staining and immune detection are as follows: PGRP-SA (2.0 μg and 200 ng), GNBP1 (1.5 μg and 300 ng), GNBP3 (1.5 μg and 300 ng), proModSP* (1.5 μg and 200 ng), procSP42 (1.5 μg and 150 ng), procSP48 (1.5 μg and 100 ng), proGrass (2.0 μg and 120 ng), proPsh (1.0 μg and 125 ng), proHayan-PA (1.5 μg and 100 ng), proHayan-PB (1.5 μg and 100 ng), proSPE (1.0 μg and 200 ng), proMP1 (1.5 μg and 200 ng), proSer7 (1.5 μg and 100 ng), proSp7 (1.5 μg and 100 ng), procSP5 (1.5 μg and 200 ng), procSP11 (1.5 μg and 200 ng), Pr1A (1.2 μg and 300 ng), proSpz (1.5 μg and 200 ng), PPO1 (1.5 μg and 300 ng), PPO2 (1.5 μg and 600 ng), and Nec (1.5 μg and 200 ng).

To gain biochemical insights into the immune protease cascade in *Drosophila*, we expressed genetically identified cascade members (ModSP, Grass, Psh, Hayan isoforms PA and PB, SPE, and Sp7), orthologs with expression patterns similar to the identified members (cSP48 and MP1), and some orthologs with relative low or stage-specific expression patterns (cSP42, Ser7, cSP5, and cSP11) as zymogens with distinct epitope tags (Fig. 1) based on ranks of their orthologs in the known cascades (fig. S1) to facilitate their cleavage product detection in subsequent in vitro reconstitution experiments. The recombinant PRRs (PGRP-SA, GNBP1, and GNBP3), negative regulator (Nec), system outputs (PPO1, PPO2, and proSpz), and *Metarhizium anisopliae* subtilisin-like protease Pr1A were also expressed and purified for functional analysis (Fig. 1).

### cSP48 acts between ModSP and Grass

In *Drosophila*, the initiating protease ModSP integrates signals from PRRs and conveys them to Grass, but it does not directly cleave the Grass zymogen (*16*), indicating the existence of an intermediate serine protease. Biochemical studies in *Manduca* have shown that Ms-HP14 (an ortholog of ModSP) activates Ms-HP21 (*37*), which, in turn, activates Ms-HP5 (an ortholog of Grass) (*11*). This result prompted us to investigate the role of *Drosophila* cSP48 and cSP42, the two *Drosophila* orthologs of Ms-HP21. cSP48 exhibits a high expression level comparable to that of ModSP and Grass, whereas cSP42 is poorly expressed (fig. S1).

**Fig. 2.**
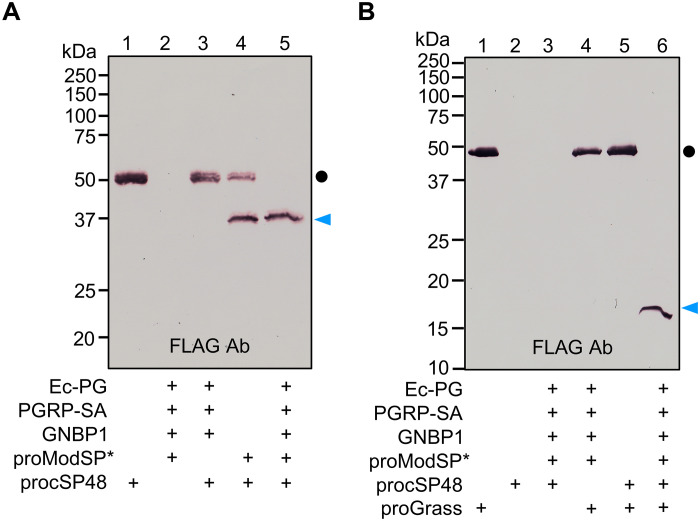
Sequential activation of procSP48 and proGrass upon proModSP autoactivation. (**A**) ProcSP48 activation. Purified procSP48 (1 μg) was incubated with *E. coli* peptidoglycan (1 μg), PGRP-SA (500 ng), GNBP1 (500 ng), proModSP* (500 ng), and buffer A (to 25 μl) for 2 hours at 37°C. The reaction mixture and controls were separated by 10% SDS-PAGE under reducing conditions and detected by immunoblotting using anti-FLAG antibody. Sizes and positions of the *M*_r_ markers are indicated. The cSP48 precursor and catalytic domain are marked with a dot and an arrowhead, respectively. (**B**) ProGrass activation. Purified proGrass (1 μg) was incubated with *E. coli* peptidoglycan (1 μg), PGRP-SA (500 ng), GNBP1 (500 ng), proModSP* (500 ng), procSP48 (100 ng), and buffer A (to 25 μl) for 2 hours at 37°C. The reaction mixture and controls were subjected to 12% SDS-PAGE under reducing conditions and detected by immunoblotting using anti-FLAG antibody. The small amount of procSP48 added in the reaction was hardly detected by the antibody, although it has a FLAG tag at the C terminus. Sizes and positions of the *M*_r_ markers are indicated. The Grass precursor and light chain produced after activation cleavage are marked with a dot and an arrowhead, respectively.

We first optimized conditions for proModSP autoactivation and tested ModSP activation of cSP48 and cSP42 zymogens. Consistent with previous results (*16*), proModSP experienced a high level of autoproteolysis during preparation (Fig. 1B). To facilitate the detection of cleavage products, we isolated the recombinant ModSP containing only the zymogen form from a fresh batch of conditioned medium through rapid purification (fig. S2A). The one containing a small amount of the activated form will be referred to as proModSP* from here on to distinguish it from the zymogen-only form (proModSP). Autoactivation occurred after proModSP was incubated with PGRP-SA, GNBP1, and insoluble Lys-PG from *Staphylococcus aureus* (Sa-PG) (fig. S2B, lane 8). Substitution of Sa-PG with soluble DAP-PG from *Escherichia coli* (Ec-PG) induced a similar response (fig. S2B, lane 9). The prevailing belief is that the Toll pathway is primarily activated by Lys-PG in *Drosophila* (*48*). The finding that Ec-PG also contributes to the activation of proModSP seems to align with the partial activation of Toll signaling (*48*–*51*). Similar level of autoactivation was also observed in the presence of β-1,3-glucan (curdlan) and GNBP3 (fig. S2B, lane 12). We noticed that the level of proModSP activation was substantially lower when compared with that of Tm-MSP (*28*) and Ms-HP14 (*33*), whose precursors were isolated directly from hemolymph. Substitution with the recombinant Ms-proHP14 impeded the autoactivation (*34*). This difference could be attributed to variances in posttranslational modifications between the recombinant protein and its natural counterpart, hindering the recombinant protein from achieving a high level of autoproteolysis in in vitro reconstitution experiments. Because proModSP* contains a small amount of activated form, we used it in the following experiments for convenience. After incubation with proModSP*, half of the 50-kDa procSP48 was converted to a 37-kDa C-terminal fragment recognized by the anti-FLAG antibody (Fig. 2A, lane 4). Complete conversion was achieved by further activating proModSP* with Ec-PG, PGRP-SA, and GNBP1 (Fig. 2A, lane 5). In the absence of dithiothreitol (DTT), the two chains of cSP48 remained attached and migrated to the 45-kDa position (fig. S3A, lane 5), slower than the procSP48 band (fig. S3A, lane 1). The uncleaved and unreduced cSP48 is more compact than the cleaved form attached by an interchain disulfide bond. In contrast, active ModSP was unable to cleave procSP42 under the same conditions (fig. S3B, left). In the absence of DTT, procSP42 migrated as a series of bands larger than 75 kDa (fig. S3B, right), indicating that dimer, tetramer, and oligomer formation may have hindered its activation by ModSP.

We next investigated the possible role of cSP48 as a Grass activator. After incubation with ModSP-activated cSP48, the 49-kDa Grass zymogen was completely processed, with its 17-kDa N-terminal fragment recognized by the anti-FLAG antibody (Fig. 2B, lane 6). ModSP, in the absence of cSP48, did not cleave proGrass (Fig. 2B, lane 4). These results indicated that cSP48 activated by ModSP was responsible for the observed proGrass cleavage. Under nonreducing conditions, the two chains of activated Grass remained attached and migrated slower than proGrass did (fig. S3C). Although the C terminus of cSP48 also contains a FLAG tag, no band was detected in lanes 2 and 3, due to a limited amount of cSP48 added, which was below the detection limit of the anti-FLAG antibody (Fig. 2B and fig. S3C). Collectively, these experiments demonstrated that cSP48, not cSP42, can be activated by ModSP, and subsequently activates proGrass to form a part of the proteolytic cascade.

### Psh and Hayan (-PA and -PB) integrate signals from Grass and microbial proteases

**Fig. 3.**
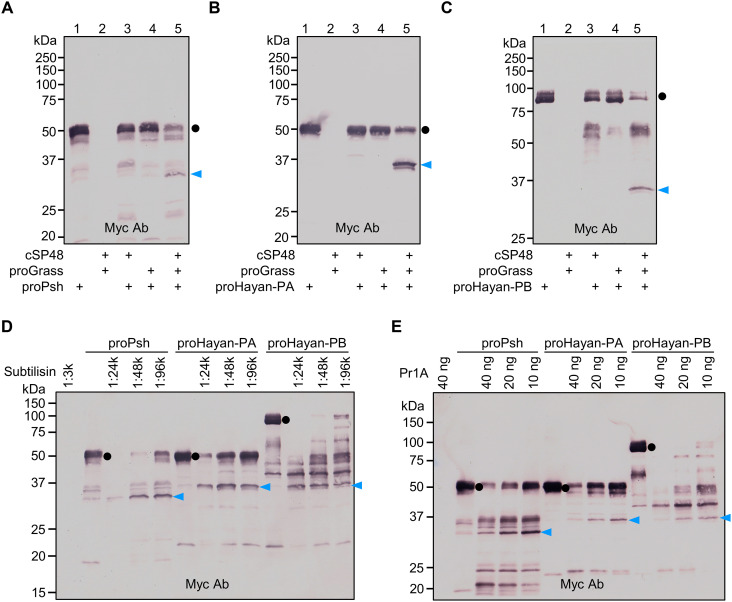
Psh and two isoforms of Hayan integrate signals from Grass and microbial proteases. (**A** to **C**) Purified proPsh (400 ng) (A) or proHayan-PA (400 ng) (B) or proHayan-PB (400 ng) (C) was incubated with active cSP48 (100 ng), proGrass (500 ng), and buffer A (to 25 μl) for 1 hour at 37°C. Active cSP48 was produced in a mixture of *E. coli* peptidoglycan (1 μg), PGRP-SA (500 ng), GNBP1 (500 ng), proModSP* (500 ng), procSP48 (100 ng), and buffer A (to 20 μl) for 1 hour at 37°C. (**D** and **E**) Purified proPsh (500 ng) or proHayan-PA (500 ng) or proHayan-PB (500 ng) was incubated with 1 μl of serially diluted subtilisin purified from *B. subtilis* (D) or various amounts of purified *M. anisopliae* Pr1A (E), and buffer A (to 25 μl) for 30 min at 29°C. The reaction mixture and controls were resolved by 10% SDS-PAGE under reducing conditions and detected by immunoblotting using anti-Myc antibody. Sizes and positions of the *M*_r_ markers are indicated. The precursors and catalytic domains of Psh, Hayan-PA, and Hayan-PB are marked with dots and arrowheads, respectively.

*Psh* and *Hayan* arose from a gene duplication, and alternative splicing can produce Hayan isoforms either similar to Psh in the case of Hayan-PA or unique to Hayan in the case of Hayan-PB and -PD (*6*). All three isoforms of Hayan contain identical clip and catalytic domains. Isoforms B and D are identical in amino acid sequence, containing a 259-residue insert located between the two domains. Although epistatic analyses suggest that Psh and Hayan function downstream of ModSP and upstream of SPE (*6*), their precise positions in the cascade remain uncertain. Taking into consideration that Psh and Hayan closely cluster with Ms-HP6 (fig. S1) and proHP6 is activated by Ms-HP5 (*11*), an ortholog of Grass, it is likely that Psh and Hayan act downstream of Grass. To test whether Grass can directly activate proPsh, proHayan-PA, or proHayan-PB, we performed cleavage and immunoblot analyses. ProPsh alone was detected as a 50-kDa band by anti-Myc antibody (Fig. 3A, lane 1). After incubation with cSP48-activated Grass, intensity of the proPsh band decreased and a new band appeared at the 32-kDa position (Fig. 3A, lane 5), consistent with the expected size of its carboxyl-terminal protease domain after cleavage activation. In the absence of DTT, the cleaved Psh migrated to the 45-kDa position (fig. S4A, lane 5), indicating the association of its clip and protease domains via an interchain disulfide bond. Similarly, upon incubation with cSP48-activated Grass, 50-kDa proHayan-PA and 90-kDa proHayan-PB underwent proteolytic cleavage to release a 36-kDa band (Fig. 3, B and C). This band corresponds to the size of their common catalytic domain. Under nonreducing conditions, the two chains of activated Hayan-PA remained attached, migrating at a slower rate than proHayan-PA (fig. S4B). Activated Hayan-PB migrated to a position greater than 37 kDa (fig. S4C, lane 5), indicating the linkage of the N-terminal fragment, but it is difficult to distinguish it from the other cleavage intermediates. Together, these experiments demonstrate that the Grass cleaved by cSP48 is proteolytically active, and the active Grass can further process proPsh, proHayan-PA, and proHayan-PB.

The Psh precursor may also act as a sensor for Toll pathway activation (*15*, *19*). While the original hypothesis was the zymogen can be directly activated by the fungal S8A protease Pr1A (*15*), a subsequent study showed that proPsh can detect proteases secreted by a wide range of pathogens through a bait region (*19*). Cleavage in this region constitutes the first step of a sequential activation process, leading to Psh maturation through additional cleavage by an endogenous cysteine protease, cathepsin 26–29-p (*19*). One exception is subtilisin, an S8A serine protease from *Bacillus subtilis* that cleaves proPsh at His^143^ to directly activate Psh. Despite the paralogous relationship, it is unclear whether microbial proteases can also activate Hayan. Therefore, we investigated the potential of *B. subtilis* subtilisin and *M. anisopliae* Pr1A to activate proHayan-PA and -PB, with proPsh included as a positive control. When incubated with the serially diluted subtilisin or Pr1A, proPsh, proHayan-PA, and proHayan-PB exhibited similar hydrolysis profiles under reducing (Fig. 3, D and E) and nonreducing conditions (fig. S4, D and E), yielding stable products similar to those generated by Grass. To determine whether cleavage activation occurred next to the predicted His, the hydrolysis products were subjected to SDS-PAGE and in-gel trypsin digestion followed by liquid chromatography tandem mass spectrometry (LC-MS/MS) analysis. We identified three peptides, (R^135^)SGNQLVIH^143^, (R^116^)SGGKPLTVH^125^, and (R^375^)TGGKPLTVH^384^, corresponding to that generated by cleavage after the predicted His residue of Psh, Hayan-PA, and Hayan-PB, respectively (fig. S5, A to C). The relative abundances of these peptides were substantially higher in the Pr1A- or subtilisin-treated groups than the control group of zymogens only (fig. S5, D to F). This result indicates that both Pr1A and subtilisin are capable of cleaving next to the His residue. Pr1A is a crucial cuticle-degrading enzyme secreted by entomopathogenic fungi *Beauveria bassiana* and *M. anisopliae*. Previous research indicated that incubation with the cell-free culture medium of *B. bassiana* failed to release the expected N-terminal extremity of the catalytic domain of Psh (*19*). This might be due to the low level of Pr1A in the conditioned medium of *B. bassiana*, as another study reported that when using a basal medium containing 1% (w/v) insect cuticle as the sole source of carbon and nitrogen, Pr1A activity was detected at a low level at 12 hours after inoculation with *M. anisopliae* and reached its maximum at 48 hours (*52*).

These experiments suggest that Psh, Hayan-PA, and Hayan-PB can all be activated by the same upstream proteases, including Grass and some microbial proteases. Moreover, both the *B. subtilis* subtilisin and the fungal protease Pr1A can directly activate the three proteases without participation of cathepsin 26–29-p from the host.

### Cleavage activation of putative terminal proteases by Psh or Hayan (-PA and -PB)

**Fig. 4.**
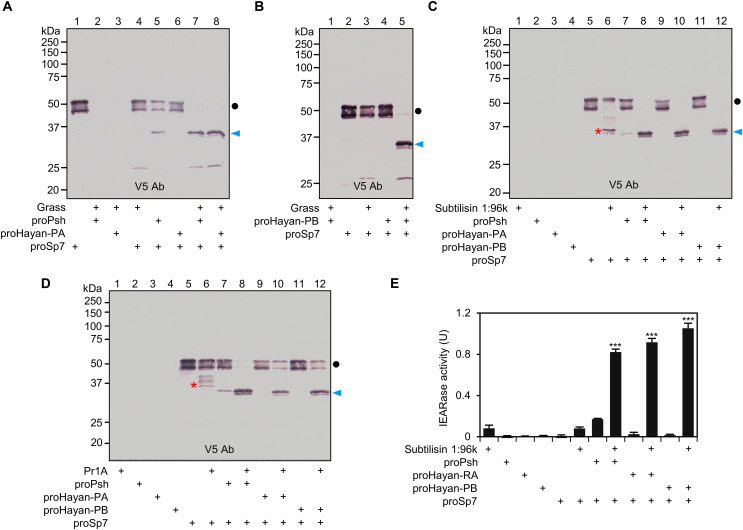
Cleavage activation of proSp7 by Psh, Hayan-PA, and Hayan-PB. Purified proSp7 (600 ng) was incubated with Grass (100 ng), proPsh (500 ng) or proHayan-PA (500 ng) (**A**) or proHayan-PB (500 ng) (**B**), and buffer A (to 25 μl) for 1 hour at 37°C. Active Grass was produced in a mixture of *E. coli* peptidoglycan (1 μg), PGRP-SA (500 ng), GNBP1 (500 ng), proModSP* (300 ng), procSP48 (100 ng), proGrass (100 ng), and buffer A (to 20 μl) for 1 hour at 37°C. In two other sets of reactions, purified proSp7 (600 ng) was incubated with 1 μl of 1:96,000 diluted subtilisin (**C**) or 20 ng of purified Pr1A (**D**), proPsh (500 ng) or proHayan-PA (500 ng) or proHayan-PB (500 ng), and buffer A (to 25 μl) for 30 min at 37°C. The reaction mixture and controls were subjected to 10% SDS-PAGE under reducing conditions and detected by immunoblotting using anti-V5 antibody. Sizes and positions of the *M*_r_ markers are indicated. The precursor and catalytic domain of Sp7 are marked with dots and arrowheads, respectively. Products from proSp7 cleaved by subtilisin or Pr1A are marked with asterisks. (**E**) Amidase activity of Sp7 activated by Psh, Hayan-PA, and Hayan-PB. In the duplicate experiment (C), amidase activities in the reaction and control mixtures were determined in 150 μl of 50 μM IEAR*p*NA and plotted as mean ± SD (*n* = 3). Statistical significance was calculated with one-way ANOVA with Tukey’s multiple comparisons test, ***P < 0.001.

Terminal proteases in the melanization and Toll pathways are characterized by their ability to process PPO or proSpz. In *Drosophila*, two terminal proteases have been identified so far, namely, Sp7 and SPE. However, their activating enzymes remain unclear. We conducted immunoblot analyses using anti-V5 antibody to investigate the activation mechanisms of Sp7, SPE, and their paralogs cSP5, cSP11, MP1, and Ser7. Recombinant proSp7 migrated as a doublet with apparent molecular masses of 48 and 52 kDa (Fig. 1B), likely due to differences in glycosylation as its sequence contains 10 predicted glycosylation sites (*21*). After incubation with Grass-activated Psh, Hayan-PA, or Hayan-PB, the proSp7 doublet was fully converted to a doublet around 35 kDa (Fig. 4, A and B), consistent with the expected size of the C-terminal protease domain after cleavage activation. In the control mixture of proPsh and proSp7, the decreased intensity of proSp7 doublet and a faint band at the size of Sp7 protease domain was observed (Fig. 4A, lane 5), due to a trace amount of active Psh in the proPsh preparation. In the absence of DTT, the two chains of activated Sp7 remained attached and migrated slower than proSp7 did (fig. S6, A and B). Similar proteolytic cleavage of proSp7 was observed under both reducing and nonreducing conditions when active Grass was replaced with subtilisin (Fig. 4C and fig. S6C) or Pr1A (Fig. 4D and fig. S6D). To confirm the activation of proSp7 by Psh, Hayan-PA or Hayan-PB, we measured the amidase activity of Sp7 using the chromogenic substrate IEAR*p*NA. A significant increase in activity was observed from Sp7 that was activated by Psh, Hayan-PA, or Hayan-PB (Fig. 4E), thus confirming activation of proSp7. Collectively, these experiments demonstrate that the active Psh, Hayan-PA, and Hayan-PB generated by Grass, subtilisin, or Pr1A are active against proSp7, generating active Sp7, a PPO1 activating enzyme (*20*, *21*). We then tested whether active Psh, Hayan-PA, and Hayan-PB were able to process procSP5 and procSP11 (fig. S7), paralogs of Sp7. Purified procSP5 contains two bands, a 60-kDa minor band corresponding to the size of cSP5 zymogen, and a 40-kDa cleavage product (fig. S7A). We found in the sequence of cSP5 a furin processing site (R^117^FKR^120^) between the clip and protease domains, whose cleavage in Sf9 cells may be responsible for the major band. The 60-kDa band somehow reduced its intensity in the reactions containing Psh, Hayan-PA, or Hayan-PB but no decrease was observed for the 40-kDa form (fig. S7, A and B). ProcSP11 was also not activated by Psh, Hayan-PA, or Hayan-PB (fig. S7C).

**Fig. 5.**
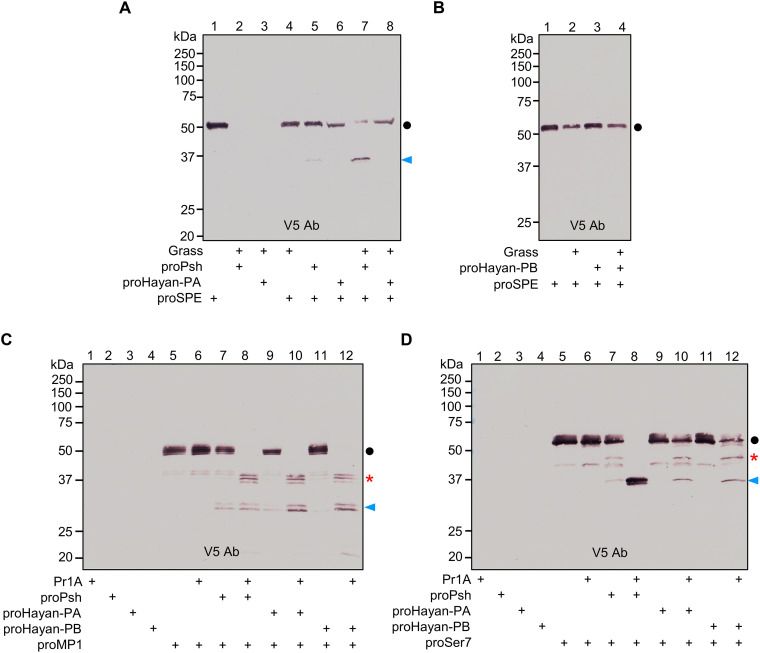
Cleavage activation of proSPE, proMP1, and proSer7. Purified proSPE (600 ng) was incubated with Grass (100 ng), proPsh or proHayan-PA (500 ng) (**A**) or proHayan-PB (500 ng) (**B**), and buffer A (to 25 μl) for 1 hour at 37°C. Active Grass was produced as described in Fig. 4 legend. The precursor and catalytic domain of SPE are marked with dots and arrowheads, respectively. In two other sets of reactions, the purified proMP1 (500 ng) (**C**) or proSer7 (500 ng) (**D**) was incubated with purified *M. anisopliae* Pr1A (20 ng), proPsh (500 ng) or proHayan-PA (500 ng) or proHayan-PB (500 ng), and buffer A (to 25 μl) for 30 min at 37°C. The precursors, catalytic domains, and unexpected cleavage products of MP1 and Ser7 are marked with dots, arrowheads, and asterisks, respectively. The reaction mixture and controls were resolved by 10% SDS-PAGE under reducing conditions and detected by immunoblotting using anti-V5 antibody. Sizes and positions of the *M*_r_ markers are indicated.

We then carried out experiments to identify proteins capable of activating SPE, i.e., Spz processing enzyme. Active Psh, but not Hayan-PA or Hayan-PB, can cleave proSPE (Fig. 5, A and B). When Grass-activated Psh was mixed with proSPE, the proSPE band decreased its intensity, and a 36-kDa product corresponding to the catalytic domain of SPE was produced (Fig. 5A, lane 7). The 36-kDa band was also detected upon incubation with proPsh (Fig. 5A, lane 5) which, again, suggests the presence of trace amount of active Psh in the proPsh preparation. Under nonreducing conditions, the activated SPE migrated slower than proSPE did (fig. S8A), indicating the linkage of the clip domain and the protease domain by an interchain disulfide bond. In contrast, the 36-kDa band did not appear after treatment of proSPE with Grass-activated Hayan-PA or -PB (Fig. 5A, lane 8, and Fig. 5B, lane 4). Although there is genetic evidence suggesting that Psh and Hayan redundantly regulate the Toll pathway (*6*), our experiments indicate that Psh and Hayan (-PA and -PB) may regulate activation of the Toll pathway differently. Given that there could be other proteases that may have the ability to cleave proSpz (*6*, *22*, *23*), we extended our investigation to explore the cleavage activation of proMP1 and proSer7, close paralogs of SPE. The 50-kDa proMP1 can be cleaved by Pr1A-generated active Psh, Hayan-PA, and Hayan-PB (Fig. 5C), resulting in the formation of two sets of cleavage products recognized by the C-terminal tag antibody. Under nonreducing conditions, a set of faint bands migrated slower than proMP1 did, representing the active form of MP1 (fig. S8B). Similarly, two sets of cleavage products were observed when proMP1 was incubated with active Psh, Hayan-PA, and Hayan-PB generated by Grass (fig. S8C). To identify which band corresponds to the product cleaved at the canonical site, the proMP1 cleaved by Pr1A-generated Psh was subjected to SDS-PAGE and in-gel Glu-C endopeptidase digestion followed by LC-MS/MS analysis. We did not detect any peptides containing the canonical cleavage site in either of these two bands. Instead, we detected a peptide (R^108^)SGTKLLPMAPNCGE^122^, positioned upstream of the canonical cleavage site of MP1 (fig. S9). This peptide is present at a high level in the 37-kDa gel slice but barely detectable in the 30-kDa protein sample, suggesting that the 30-kDa band represents the catalytic domain of MP1 produced by cleavage at the correct bond. ProSer7 migrated to a position of 55 kDa and was also processed by Pr1A- (Fig. 5D) and Grass-generated (fig. S8D) active Psh, Hayan-PA, and Hayan-PB. The proSer7 activation led to the formation of two sets of cleavage products. One migrated to the 37-kDa position, consistent with the size of the C-terminal protease domain, while the other migrated to 45 kDa, indicating that cleavage occurred before the proteolytic activation site. In the absence of DTT, the cleavage products migrated faster than the proSer7 band (fig. S8, E and F), suggesting that additional cleavage in the linker between the clip and protease domains caused a loss of N-terminal fragment.

In summary, our findings indicate that Psh and Hayan (-PA and -PB) have partially overlapping roles in processing terminal proteases and their paralogs. Specifically, Psh cleaves proSp7, proSPE, proMP1, and proSer7, while Hayan (-PA and -PB) only activates proSp7, proMP1, and proSer7.

### Cleavage activation of proSpz, PPO1, and PPO2

The processing of terminal proteases and their paralogs by Psh or Hayan (-PA and -PB) led to experiments to test whether cleaved Sp7, SPE, MP1, and Ser7 are active against proSpz, and two PPOs (PPO1 and PPO2) responsible for melanization (*53*, *54*).

**Fig. 6.**
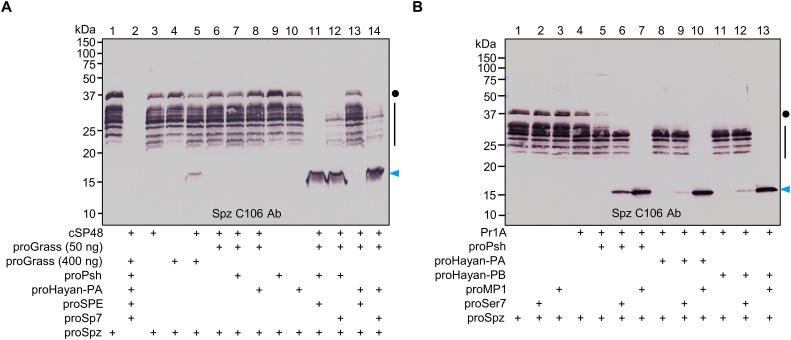
Cleavage activation of proSpz by SPE, Sp7, MP1, and Ser7. (**A**) Purified proSpz (400 ng) was incubated with cSP48 (100 ng), proGrass (50 or 400 ng), proPsh (500 ng) or proHayan-PA (500 ng), proSPE (500 ng) or proSp7 (500 ng), and buffer A (to 25 μl) for 1 hour at 37°C. Active cSP48 was produced in a mixture of *E. coli* peptidoglycan (1 μg), PGRP-SA (500 ng), GNBP1 (500 ng), proModSP* (300 ng), procSP48 (100 ng), and buffer A (to 20 μl) for 1 hour at 37°C. (**B**) Purified proSpz (400 ng) was incubated with Pr1A (20 ng), proPsh (500 ng) or proHayan-PA (500 ng) or proHayan-PB (500 ng), proMP1 (500 ng) or proSer7 (500 ng), and buffer A (to 25 μl) for 1 hour at 37°C. The reaction mixture and controls were subjected to 12% SDS-PAGE under reducing conditions and immunoblot analyses using anti-Spz C106 antibody. Sizes and positions of the *M*_r_ markers are indicated. The Spz precursor, partially processed Spz, and the cystine-knot domain are marked with dots, vertical lines, and arrowheads, respectively.

The canonical Toll receptor (Toll-1) is activated by the cytokine Spz-1, also known as Spz, one of the six Spz genes found in *Drosophila*. We first performed immunoblot analyses using the anti-Spz C106 antibody (*55*) to examine possible cleavage of proSpz by the four active proteases. After incubation with the SPE (Fig. 6A, lane 11), Sp7 (Fig. 6A, lanes 12 and 14), and MP1 (Fig. 6B, lanes 7, 10, and 13), almost all the proSpz was converted to a 16-kDa band corresponding to the activated cystine-knot domain. In addition, Grass (Fig. 6A, lane 5) and Ser7 (Fig. 6B, lanes 6, 9, and 12) were also able to process proSpz and generate a faint band at the same position. In the absence of DTT, this protein migrated to a position around 22 kDa (fig. S10), consistent with the size of a disulfide-linked dimer. Previous research showed that *SPE*-deficient flies exhibit a similar level of Toll pathway activation as control flies upon challenge with *B. subtilis* (*22*), indicating that unidentified protease(s) can process proSpz besides SPE. Sp7 (PPO1 activating protease) and SPE (proSpz processing enzyme) are currently the only genetically characterized terminal proteases. To further understand their roles in the *B. subtilis* infection model, we generated double mutants for *Sp7* and *SPE*. Our results showed that *Sp7^SK6^*, *SPE^SK6^* double mutants exhibit slightly lower levels of *Drs* mRNA compared with wild-type flies after *B. subtilis* infection (fig. S11). The quasi–wild-type level of Toll activity upon septic injury with *B. subtilis* might be attributed to MP1, Ser7, and Grass in the hemolymph. Of note, we observed a significant increase in *Drs* mRNA levels in *Sp7* mutants following *B. subtilis* infection compared to wild-type flies, likely due to compensatory up-regulation caused by defects in the melanization response (*6*, *53*). This observation further highlights the limitations of genetic studies in characterizing genes with redundant functions. Collectively, these experiments suggest that, in addition to SPE, four other clip-domain proteases Sp7, MP1, Ser7, and Grass may also function in proSpz activation.

**Fig. 7.**
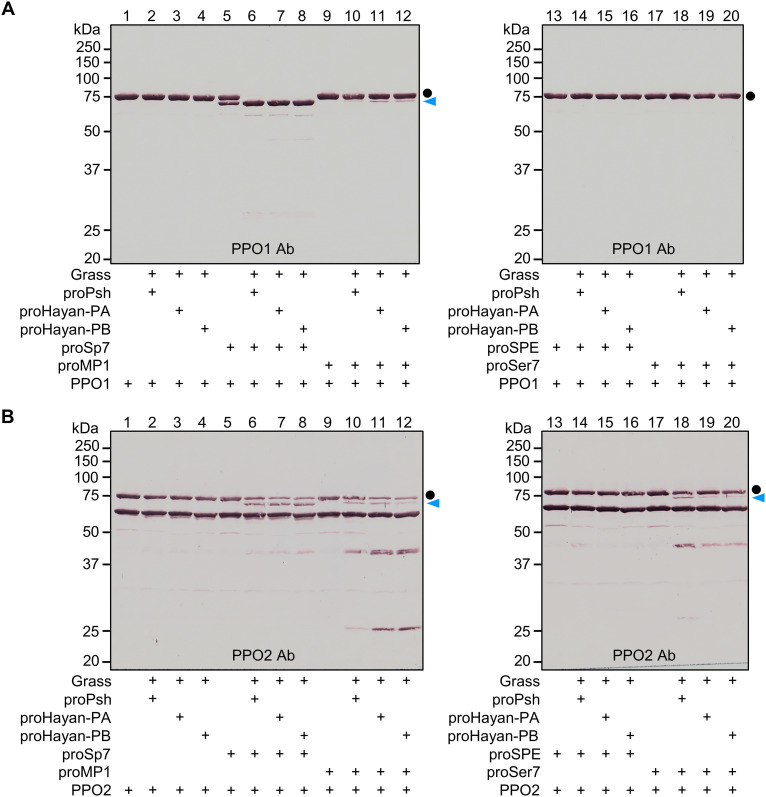
Cleavage activation of PPO1 and PPO2. Purified PPO1 (600 ng) (**A**) or PPO2 (1600 ng) (**B**) was incubated with Grass (100 ng), proPsh (500 ng) or proHayan-PA (500 ng) or proHayan-PB (500 ng), proSp7 (500 ng) or proMP1 (500 ng) or proSPE (500 ng) or proSer7 (500 ng), and buffer A (to 25 μl) for 45 min at 25°C. Active Grass was produced as described in Fig. 4 legend. The reaction mixture and controls were subjected to 10% SDS-PAGE under reducing conditions and detected by immunoblotting using PPO1 or PPO2 antibodies. Sizes and positions of the *M*_r_ markers are indicated. The precursors and active forms of PPO1 and PPO2 are marked with dots and arrowheads, respectively.

To explore possible involvement of these proteases in PPO activation, we incubated purified PPO1 (Fig. 7A) or PPO2 (Fig. 7B) with cleaved Sp7, SPE, MP1, or Ser7, and then performed immunoblot analyses using either anti-PPO1 or PPO2 antibody. Incubation with proSp7 resulted in the cleavage of a portion of 75-kDa PPO1 and appearance of a new band around 70-kDa corresponding to active PO1 (Fig. 7A, lane 5), likely due to a trace amount of active Sp7 in the proSp7 preparation. After Sp7 was generated by Psh, Hayan-PA, or Hayan-PB, all the 75-kDa PPO1 was converted to the 70-kDa active PO1 (Fig. 7A, lanes 6 to 8). In contrast, MP1 was only able to convert a small amount of PPO1 to its active form (Fig. 7A, lanes 10 to 12), while SPE and Ser7 did not cleave PPO1 (Fig. 7A, lanes 13 to 20). Similarly, Sp7 was found to be the primary activating enzyme for PPO2, converting the 75-kDa PPO2 into the 70-kDa active PO2 (Fig. 7B, lanes 6 to 8). MP1 (Fig. 7B, lanes 10 to 12) and Ser7 (Fig. 7B, lanes 18 to 20) were also observed to process PPO2 to a lesser extent, while SPE was unable to process PPO2 (Fig. 7B, lanes 13 to 16). Despite a previous study indicating that the catalytic domain of Hayan can directly activate PPO1 (*5*), our experiments demonstrate that neither Hayan-PA nor Hayan-PB can directly activate PPO1 (Fig. 7A, lanes 3 and 4) or PPO2 (Fig. 7B, lanes 3 and 4), suggesting that the clip domain may play a crucial role in binding interactions with other proteins to ensure the hierarchical activation of the protease cascades.

### Inhibition of ModSP and Grass by Nec

**Fig. 8.**
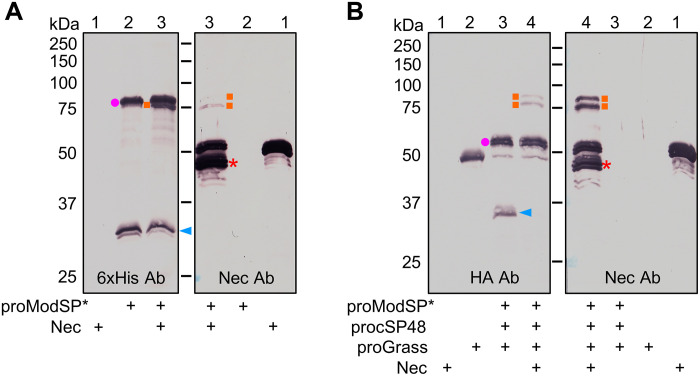
Formation of covalent complexes of Nec and ModSP or Grass. (**A**) Formation of SDS-stable complexes of Nec and ModSP. Purified Nec (800 ng) was incubated with proModSP* (1 μg), and buffer A (to 25 μl) for 1 hour at 37°C. The reaction mixture and controls were separated by 10% SDS-PAGE under reducing conditions and detected by immunoblotting using 6×His (left) or Nec (right) antibodies. Sizes and positions of the *M*_r_ markers are indicated. The ModSP zymogen, catalytic domain of ModSP, ModSP-Nec complex, and cleaved Nec are marked with a dot, an arrowhead, squares, and an asterisk, respectively. (**B**) Formation of SDS-stable complexes of Nec and Grass. Active Grass was produced in a mixture of proModSP* (500 ng), procSP48 (100 ng), proGrass (800 ng), and buffer A (to 23 μl) for 1 hour at 37°C. Then, Nec (800 ng, 2 μl) was added to the mixture and incubated for 1 hour at 37°C. The reaction mixture and controls were subjected to 10% SDS-PAGE under reducing conditions and detected by immunoblotting using HA (left) or Nec (right) antibodies. Sizes and positions of the *M*_r_ markers are indicated. The cSP48 zymogen, catalytic domain of Grass, Grass-Nec complex, and cleaved Nec are marked with a dot, an arrowhead, squares, and an asterisk, respectively.

After successful reconstitution of the protease network in vitro, we turned our attention to addressing the long-standing question on the target of Nec. Genetic evidence indicated that mutations in *psh* suppressed all *nec* phenotypes (*25*), suggesting that Nec acts on Psh or its upstream proteases. As Psh is activated through the sequential activation of ModSP, cSP48, and Grass, we used specific antibodies to determine whether Nec can form complexes with one or more of these proteases. When proModSP*, which contained a small amount of active ModSP, was incubated with Nec and visualized by anti-6×His antibody, the band representing the 31-kDa catalytic domain became less intense, and a band at 75 kDa appeared (Fig. 8A, left lane 3). Two bands were detected in the high *M*_r_ region when probed with the Nec antibody (Fig. 8A, right lane 3): one at 75 kDa, which corresponded to the one recognized by the anti-6×His antibody, and the other at 80 kDa, which became visible after grayscale adjustment (fig. S12, right lane 3). As the proModSP also migrated around the 80-kDa position, the 80-kDa ModSP-Nec complex cannot be distinguished from proModSP with the anti-6×His antibody (Fig. 8A, left). The formation of two complexes with distinct sizes by a single protease domain and a serpin is unusual, implying that the constituent proteins of these complexes may have undergone a differential modification. Previous studies indicated that the N-terminal polyglutamine extension of Nec is released after immune challenge (*56*). To test whether the cleavage also occurs in this condition, the solution samples were subjected to matrix-assisted laser desorption/ionization–time-of-flight (MALDI-TOF) and LC-MS/MS analyses. When incubated with proModSP*, the peptides covering the first 49-residue region of the mature Nec was detected by LC-MS/MS (fig. S13A). MALDI-TOF did not detect a peak that corresponds to the *M*_r_ of the initial 49 amino acids (5993 Da) (fig. S14A), likely due to additional cleavage (*56*). We did not detect cSP48 in complex with Nec forms using the FLAG antibody to the C-terminal protease domain of cSP48 (fig. S15A). When active Grass was incubated with Nec and visualized by anti-HA antibody, the band representing the 34-kDa catalytic domain of Grass disappeared, and two high *M*_r_ complexes were detected (Fig. 8B, left lane 4). Meanwhile, we observed two intense bands using anti-Nec antibody at 80 and 75 kDa (Fig. 8B, right lane 4), suggesting that each of the two bands represents the complexes of ModSP-Nec and Grass-Nec. LC-MS/MS analysis of the two bands confirmed the presence of ModSP and Grass catalytic domains (fig. S16). Consistently, MALDI-TOF analysis revealed a 4316-Da peptide, which is notably more abundant in reaction of Nec and Grass (fig. S14A) and identical in mass to the C-terminal peptide (S^438^LPP…M^ox^SV^476^) released after cleavage at the P1 Leu^437^. The cleavage site was further supported by the detection of S^438^LPP…FVF^454^ in the reaction of Grass (fig. S13C). Together, these results indicated that Nec not only inhibits ModSP, a chymoelastase-like enzyme that prefers P1 Leu, but also inhibits Grass, a trypsin-like enzyme that prefers P1 Arg. Subsequent immunoblot analyses using anti-Myc antibody did not detect a complex of Psh and Nec (fig. S15B). In the presence of excess Nec, ModSP failed to cleave its substrate procSP48 (fig. S17A) while Grass did not cleave its substrate Psh and Hayan-PA (fig. S17B). Therefore, Nec controls Toll and PPO activation by inhibiting both ModSP and Grass.

## DISCUSSION

Insects rely on innate immune responses including melanization and antimicrobial peptide synthesis to defend against invading pathogens. These reactions involve integrated protease cascade pathways, wherein the recognition of non- or altered self triggers the autoactivation of an initiating protease (*16*, *28*, *32*, *33*). The initiation signals are relayed through the activation of multiple downstream S1A serine proteases. Specific serpins inhibit some of these proteases to modulate the immune responses in a temporal and spatial manner, as the products of immune responses can harm host insects as well (*57*).

**Fig. 9.**
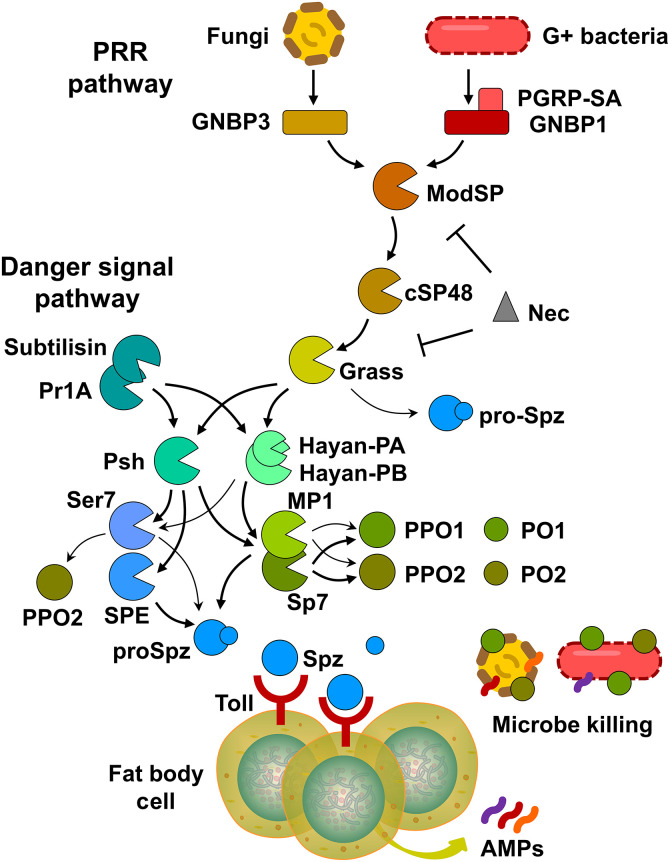
An improved model of the serine protease system regulating Toll signaling and PPO activation. Pathogen invasion initiates the activation of an extracellular serine protease network through recognition of microbial molecular patterns as well as virulent factors from the pathogens. These patterns are recognized by PRRs, such as PGRP-SA and GNBP1 (for Gram-positive bacteria mainly) or GNBP3 (for fungi), triggering the sequential activation of ModSP, cSP48, Grass, and Psh/Hayan-PA/PB. Hayan-PA and Hayan-PB play a similar role in the activation of terminal proteases, both sharing a partially redundant function with Psh. Psh mediates the activation of SPE, Sp7, MP1, and Ser7, whereas the two isoforms of Hayan are only involved in the activation of Sp7, MP1, and Ser7. The activation of the terminal proteases leads to the cleavage of PPOs and proSpz, resulting in melanization response and Toll pathway activation in fat body. The activation of PPO1 and PPO2 is primarily mediated by Sp7, whereas MP1 plays a minor role in this process. Ser7 also appears to function in the activation of PPO2, albeit weakly. In addition to SPE, Sp7, MP1, Ser7, and Grass also participate in the activation of proSpz. Microbial proteases, such as subtilisin and Pr1A, can activate the melanization and Toll pathways by directly activating Psh as well as the two isoforms of Hayan. Nec negatively regulates the immune responses by inhibiting both ModSP and Grass. Arrows with thick lines represent a high degree of proteolytic cleavage and play major roles in melanization and/or Toll pathway activation, whereas arrows with thin lines represent a low level of cleavage activation and minor contributions.

Here, we reconstituted a complex serine protease network that integrates PRR and danger signaling in *Drosophila* using biochemical methods (Fig. 9). In the PRR pathway, detection of cell wall components such as peptidoglycan from bacteria or β-1,3-glucan from fungi through the PGRP-SA/GNBP1 complex or GNBP3, respectively, constitutes the crucial step in a successful host defense. Despite the prevailing belief that the Toll pathway is activated by Gram-positive bacteria and fungi, whereas the Imd pathway responds to Gram-negative bacterial infection (*48*, *58*, *59*), our data indicates that DAP-PG from Gram-negative bacteria can also trigger the autoactivation of ModSP in conjunction with PGRP-SA and GNBP1 (fig. S2). This finding is consistent with the partial activation of Toll signaling by certain Gram-negative bacteria (*48*–*51*) and the importance of peptidoglycan accessibility in bacterial sensing (*51*). The active ModSP subsequently activates cSP48, which, in turn, activates Grass (Fig. 2 and fig. S3). This branch is conserved in *Manduca*, as their respective orthologs also form a proteolytic cascade, HP14-HP21-HP5 (*11*, *37*). A future study using genetic approach is necessary to confirm the role of cSP48.

Previous bioinformatics analysis indicated that *psh* is a lineage-restricted duplication arising from an ancestral *Hayan* gene (*6*) and an insert between the clip and catalytic domains had evolved to sense pathogenic proteases (*19*). However, our results (Fig. 3 and fig. S4) indicated that this function may have preexisted in Hayan-PA and Hayan-PB. Lacking the 259-residue Hayan-PB exclusive region, Hayan-PA did not show any difference in activation by the microbial proteases or in activating downstream proteases compared to Hayan-PB. The two forms of Hayan, together with Psh, serve as a platform, integrating signals from PRR and danger signal pathways. Same as Grass, subtilisin of *B. subtilis* and Pr1A of *M. anisopliae* each directly activates Psh, Hayan-PA, and Hayan-PB. Furthermore, in comparison to Hayan-PA and -PB, Psh also activates SPE, in addition to Sp7, MP1, and Ser7 (Figs. 4 and 5 and figs. S6 and S8).

Because Psh and Hayan redundantly regulate Toll signaling, as characterized by genetic analysis (*6*), our result that two Hayan isoforms did not activate SPE (Fig. 5) suggests the existence of unidentified protease(s) downstream of Hayan that can process proSpz (*22*, *23*). We identified four other proSpz activators, namely, Sp7, MP1, Ser7, and Grass (Fig. 6 and fig. S10). MP1 and Ser7 are close paralogs of SPE. The conserved function observed within this cluster of lineage-specifically expanded genes may contribute to the robustness of the immune system. Sp7, known as the activating enzyme for PPO1, also has the ability to activate proSpz. This result is consistent with the findings reported in *Tenebrio*, where Tm-SPE was found to activate both proSpz and PPO (*28*, *30*). The functional interrelatedness of the melanization and Toll cascade pathways may be rooted in their common evolutionary origin and might reflect a common readiness or priming to face newly perceived threats. In *Manduca*, the ortholog of Grass, Ms-HP5, has also been shown to activate proSpz directly (*40*). The ability of Grass and Ms-HP5 to process proSpz is supported by the structure-based classification of clip-domain serine proteases, which places them in the terminal protease group along with Dm-Easter, Dm-SPE, Ms-HP8, and Tm-SPE (*60*, *61*). All of these terminal proteases feature a group 2 clip domain, as well as an additional loop (75-loop) in the catalytic domain.

In addition to activating proSpz, proteases capable of activating PPO are also known as terminal proteases. The *Drosophila* genome encodes three PPOs, two of them, PPO1 and PPO2, are produced by crystal cells and contribute to hemolymph melanization (*53*, *54*). Here, we show that Sp7 is the primary activating enzyme for both PPO1 and PPO2, while MP1 plays a minor role in this process (Fig. 7). The minor contribution of MP1 in PPO activation aligns with the fact that overexpression of the catalytic domain of MP1 led to the constitutive melanization phenotype (*20*), but *MP1*-deficient flies exhibit no apparent defects in PPO activation (*6*), likely due to functional redundancy. We found that Ser7 may also play a minor role in the activation of PPO2 (Fig. 7). However, because PPO2 accumulates in the crystals of crystal cells and is deployed in a later phase of infection (*53*), the minor contribution of Ser7 to the melanization response may be hard to discern through genetic approaches.

In this complex protease network, Nec negatively regulates progression of the immune responses by simultaneously inhibiting the activities of ModSP and Grass (Fig. 8 and fig. S17). With a Leu at putative P1 residue, Nec is predicted to inhibit a chymoelastase-like enzyme. In the current model, ModSP is the only protease displaying such specificity, whereas the other proteases are all trypsin-like enzyme. The inhibition of protease Grass by Nec is intriguing. Notably, Grass exhibits a unique cleavage specificity, as its natural substrates (Psh, Hayan-PA, and Hayan-PB) all harbor an unusual His residue at the activation site. Like the human α1-protease inhibitor, with a P1 Met inhibiting trypsin, chymotrypsin, and elastase (*62*), Nec also has a P2 Pro that may broaden its inhibitory selectivity. Further biochemical research is needed to characterize the role of Pro in presenting P1 Leu to the S1 pocket of Grass.

The serine protease network revealed in this study is highly similar to that of *Manduca*. In comparison, the three-step proteolytic cascade (MSP-SAE-SPE) found in *Tenebrio* (*28*, *30*) appears to be the abridged version of a complex network. A short cascade (HP14-HP21-PAP2/PAP3) (*37*, *41*) and an extended pathway (HP14-HP21-HP5-HP6-PAP1) (*11*, *38*) both lead to PPO activation in *Manduca*. The latter is conserved in *Drosophila* as ModSP-cSP48-Grass-Psh/Hayan-Sp7/MP1/Ser7. Duplication of genes encoding proteases at key steps of these pathways likely increased the diversity and plasticity of the immune response, while a short cascade may provide a quicker reaction. Aside from its role in PPO activation, Ms-PAP3 is known to expedite immune responses by activating precursors of PAP3, HP5, and noncatalytic serine protease homologs (*11*, *44*, *63*–*65*). The cross-talk between cascades, together with functional redundancy caused by gene duplication, further increases the complexity of the protease network. Overall, our research provides an improved paradigm for the study of the complex serine protease network in *Drosophila* and beyond. Phylogenetic relationships provide insights into evolutionary relatedness, enabling the identification of functionally conserved genes across taxa. Meanwhile, expression profiles allow the assessment of gene expression patterns, facilitating the selection of genes with dynamic and context-specific roles. Coupling these data with biochemical approaches will help overcome limitations in reverse genetics for resolving overlapping functions within complex pathways. Compared to exclusive expression of the catalytic domain of serine proteases, using proenzyme expression and activation to verify functionality appears to be more reliable. This is because the inclusion of regulatory domains, such as the clip domain, allows specific protein-protein interactions to occur among the pathway components. Future studies using compound mutant analysis are also necessary to gain a better understanding of this complex immune protease network.

## MATERIALS AND METHODS

### Microbial ligands and protease

Insoluble β-1,3-glucan (curdlan) from *Alcaligenes faecalis* (#C7821) and subtilisin from *Bacillus* (#P5860) were purchased from Sigma. Soluble DAP-type peptidoglycan from the Gram-negative bacterium *E. coli* K12 (Cat. tlrl-ksspgn) and insoluble Lys-type peptidoglycan from the Gram-positive bacterium *S. aureus* (Cat. tlrl-pgns2) were purchased from InvivoGen.

### Fly strains

*w1118* flies were used as wild-type control. *Sp7^SK6^* (*54*) and *SPE^SK6^* (*55*) mutant flies were described previously. The *Sp7^SK6^, SPE^SK6^* double mutants were generated by crossing *Sp7^SK6^* and *SPE^SK6^* lines performed by WellGenetics Inc. The resulting recombinant flies were verified by genomic PCR with the following primers: *Sp7^SK6^* forward: 5′-TGCCCTCGCCACCCACTC, reverse: 5′-GTCCACGTCCTTGCTGGTAT; *SPE^SK6^* forward: 5′-GTTGTACGCCCCAGCGAG, reverse: 5′-ATTCCCAGAGGGTCGTATTG. The *Sp7^SK6^, SPE^SK6^* double mutants were viable and showed no differences from the wild-type control under standard growth conditions.

### Bacteria culture and immune challenge

*B. subtilis* were cultured in Luria Broth (LB) at 37°C. Systemic infections (septic injury) were performed by pricking adults in the thorax with a thin needle previously dipped into a concentrated *B. subtilis* (OD_600_ = 10). Infected flies were subsequently maintained at 25°C for 16 h.

### Quantitative RT-PCR

For quantification of mRNA levels, whole flies were collected at 16 hours after *B. subtilis* infection. Total RNA was isolated from a minimum of 10 adult flies using TRIzol reagent (Thermo Fisher Scientific) and dissolved in RNase-free water. The single-strand cDNA, synthesized from the RNA using iScript Reverse Transcription Supermix (Bio-Rad), was used as a template for quantitative reverse transcription polymerase chain reaction (RT-PCR). The quantitative RT-PCR was performed on a CFX Connect Real-Time System (Bio-Rad) using iTaq Universal SYBR Green Supermix (Bio-Rad) according to the manufacturer’s instructions. Primers were as follows: *Drosomycin* forward primer (j1955 5′-CGTGAGAACCTTTTCCAATATGAT), reverse primer (j1956 5′-TCCCAGGACCACCAGCAT); RpL32 (i.e. Rp49) forward primer (j1957 5′-GACGCTTCAAGGGACAGTATCTG), reverse primer (j1958 5′-AAACGCGGTTCTGCATGAG).

### Modification of baculovirus vector

The plasmid pMFH6 (*66*), which encodes the signal peptide of honeybee melittin and a C-terminal 6×His tag, was modified by introducing N-terminal and/or C-terminal tags to facilitate the detection of purified recombinant proteins. Briefly, j085 (5′-AATTCATGATTACAAGGATGACGACGATAAGCATATG) and j086 (5′-TCGACATATGCTTATCGTCGTCATCCTTGTAATCATG) were phosphorylated, annealed, and inserted to the Eco RI and Sal I sites of pMFH6 to generate pMFFH6 (N-terminal FLAG tag, C-terminal 6×His tag); j087 (5′-TCGAGCAGAAGCTCATCTCTGAAGAGGATCTGCATCACCATCACCATCACTA) and j088 (5′-AGCTTAGTGATGGTGATGGTGATGCAGATCCTCTTCAGAGATGAGCTTCTGC) were phosphorylated, annealed, and inserted to the Xho I and Hin dIII sites of pMFFH6 to generate pMFFMH6 (N-terminal FLAG tag and C-terminal Myc tag followed by 6×His tag); j089 (5′-TCGAGTACCCTTACGACGTGCCTGACTACGCTCATCACCATCACCATCACTA) and j090 (5′-AGCTTAGTGATGGTGATGGTGATGAGCGTAGTCAGGCACGTCGTAAGGGTAC) were phosphorylated, annealed, and inserted to the Xho I and Hin dIII sites of pMFFH6 to generate pMFFHH6 (N-terminal FLAG tag and C-terminal HA tag followed by 6×His tag); j2007 (5′-TCGAGGGTAAGCCTATCCCTAACCCTCTCCTCGGTCTCGATTCTACGCATCACCATCACCATCACTA) and j2008 (5′-AGCTTAGTGATGGTGATGGTGATGCGTAGAATCGAGACCGAGGAGAGGGTTAGGGATAGGCTTACCC) were phosphorylated, annealed, and inserted to the Xho I and Hin dIII sites of pMFFH6 to generate pMFFVH6 (N-terminal FLAG tag and C-terminal V5 tag followed by 6×His tag); j2009 (5′-AATTCATTACCCTTACGACGTGCCTGACTACGCTCATATG) and j2010 (5′-TCGACATATGAGCGTAGTCAGGCACGTCGTAAGGGTAATG) were phosphorylated, annealed, and inserted to the Eco RI and Sal I sites of pMFH6 to generate pMHFH6 (N-terminal HA tag and C-terminal 6×His tag); j2011 (5′-TCGAGGATTACAAGGATGACGACGATAAGCATCACCATCACCATCACTA) and j2012 (5′-AGCTTAGTGATGGTGATGGTGATGCTTATCGTCGTCATCCTTGTAATCC) were phosphorylated, annealed, and inserted to the Xho I and Hind III sites of pMHFH6 to generate pMHFFH6 (N-terminal HA tag and C-terminal FLAG tag followed by 6×His tag).

### Cloning and production of recombinant proteins

The coding sequence of mature proModSP was amplified by PCR using forward primer (j1097 5′-GAATTCTATGTGACAGCTCACAGTTTG), reverse primer (j1098 5′-CTCGAGGGACCTAGTTTCCACGCTC), and the cDNA clone LD43740 (DGRC) as template. Similarly, procSP42 was amplified using forward primer j1859 (5′-CATATGGAATTCTGCGATAATGGAAC), reverse primer j1860 (5′-CTCGAGCTCGCCCCACACGAT), and the cDNA clone IP10038 (DGRC) as template; procSP48 was amplified using forward primer j1857 (5′-CATATGGAGTACTGCGACAATGG), reverse primer j1858 (5′-CTCGAGATTGCCCCACACGATG), and the cDNA clone GH06673 (DGRC) as template; proGrass was amplified using forward primer j1861 (5′-CATATGGCCCGAGCAGACTAC), reverse primer j1862 (5′-CTCGAGGCCATTGCTGGC), and the cDNA clone RH61984 (DGRC) as template; proPsh was amplified using forward primer j1867 (5′-CATATGGCCGTCACAGTGGG), reverse primer j1868 (5′-CTCGAGCACCCGATTGTCCG), and the cDNA clone GH12385 (DGRC) as template; proHayan-PA was amplified using forward primer j1869 (5′-CATATGTATGTAACAGTTGGGGAT), reverse primer j1870 (5′-CTCGAGGAAACGATTCGACGG), and the cDNA clone GH17483 (DGRC) as template; proHayan-PB was amplified using primers j1869 and j1870, and cDNA from adult flies as template, and a S376T mutation was observed compared with NP_001097020.1 [National Center for Biotechnology Information (NCBI)]; proSPE was amplified using forward primer j1871 (5′-CATATGGCGGAGATCAGCTTTG), reverse primer j1872 (5′-CTCGAGTGGCTCCAATTTCTGC), and the cDNA clone GH28857 (DGRC) as template; proMP1 was amplified using forward primer j1873 (5′-CATATGCAAGAAATCTTTGGGTAT), reverse primer j1874 (5′-CTCGAGAGCCCTAACGTTGTT), and the cDNA clone from a previous study as template (*21*); proSer7 was amplified using forward primer j1875 (5′-CATATGCAGCAATTTGGCAAAG), reverse primer j1876 (5′-CTCGAGAATGCGATTGGCTCG), and the cDNA clone GH14088 (DGRC) as template; proSp7 was amplified using forward primer j1551 (5′-CATATGCAAGGAAGTTGTAGG), reverse primer j1552 (5′-CTCGAGGGGACGAATGGTCTC), and the cDNA clone from a previous study as template (*21*); procSP5 was amplified using forward primer j1881 (5′-CATATGGAGGTCCTGCAGGA), reverse primer j1882 (5′-CTCGAGAGCCATTTTGTCCTG), and the cDNA clone RE44245 (DGRC) as template; procSP11 was amplified using forward primer j1877 (5′-CATATGCAGTACGTGAGTTGCC), reverse primer j1878 (5′-CTCGAGAGGTCGTATATTCTGCG), and the cDNA clone IP10721 (DGRC) as template; Pr1A was amplified using forward primer j2022 (5′-CATATGGCTGAGCCAGCTCCT), reverse primer j2023 (5′-CTCGAGGGCACCGTTGTAGG), and the cDNA clone pJM 969 from a previous study as template (*15*); proSpz was amplified using forward primer j1883 (5′-GAATTCAGGACAGTGCACCC), reverse primer j1884 (5′-CTCGAGCCCAGTCTTCAACGC), and the cDNA clone FI05217 (DGRC) as template; PGRP-SA was amplified using forward primer j1095 (5′-GAATTCGAAAGTCGCGTCAGCGT), reverse primer j1096 (5′-CTCGAGGGGATTTGAGAGCCAGTG), and the cDNA clone AT30827 (DGRC) as template; GNBP1 was amplified using forward primer j1099 (5′-GAATTCACAAGATCCCCACACCGACTG), reverse primer j1100 (5′-CTCGAGGTTGGCGAAGACACGAAC), and the cDNA clone LD15841 (DGRC) as template; GNBP3 was amplified using forward primer j1093 (5′-GAATTCTACCCAAGGCTAAGATC), reverse primer j1094 (5′-CTCGAGAGAGTACACCTTTAC), and the cDNA clone LP05991 (DGRC) as template. The purified products were ligated with pGEM-T vector (Promega). Following sequence validation, the plasmids PGRP-SA/pGEM-T, GNBP1/pGEM-T, GNBP3/pGEM-T, proModSP/pGEM-T, and proSpz/pGEM-T were digested using restriction enzymes Eco RI and Xho I, and the Eco RI-Xho I fragments were subcloned into the same sites in pMFH6; the plasmids procSP42/pGEM-T and procSP48/pGEM-T were digested using restriction enzymes Nde I and Xho I, and the Nde I-Xho I fragments were subcloned into the same sites in pMHFFH6; the plasmids proGrass/pGEM-T and Pr1A/pGEM-T were digested using restriction enzymes Nde I and Xho I, and the Nde I-Xho I fragments were subcloned into the same sites in pMFFHH6; the plasmids proPsh/pGEM-T, proHayan-PA/pGEM-T, and proHayan-PB/pGEM-T were digested using restriction enzymes Nde I and Xho I, and the Nde I-Xho I fragments were subcloned into the same sites in pMFFMH6; the plasmids proSPE/pGEM-T, proMP1/pGEM-T, proSer7/pGEM-T, proSp7/pGEM-T, procSP5/pGEM-T, and procSP11/pGEM-T were digested using restriction enzymes Nde I and Xho I, and the Nde I-Xho I fragments were subcloned into the same sites in pMFFVH6. The plasmid was used to generate a bacmid and high-titer viral stock for infecting Sf9 cells. The recombinant protein was enriched from 300 ml of conditioned medium using ion-exchange chromatography on a Q-Sepharose column (for procSP42, procSP48, proHayan-PA, and proHayan-PB) or a dextran sulfate–Sepharose CL-6B column (for the other proteins), followed by purification via affinity chromatography on a Ni^2+^-NTA agarose column. For Q-Sepharose chromatography, the cell-free conditioned medium obtained by centrifugation at 5,000*g* for 20 min was supplemented with 1 mM benzamidine and adjusted to pH 8.0 before applied to a Q-Sepharose column (40 ml) equilibrated in Q buffer (20 mM tris and 1 mM benzamidine, pH 8.0). After washing with 200 ml of Q buffer, a linear gradient of 0 to 1.0 M NaCl in Q buffer was used to elute the bound proteins at 1.5 ml/min for 100 min, followed by 1.0 M NaCl in Q buffer at 1.5 ml/min for 50 min. For dextran sulfate chromatography, the cell-free medium containing secreted proteins was diluted with an equal volume of deionized H_2_O, supplemented with 1 mM benzamidine and adjusted to pH 6.4 before applied to a dextran sulfate–Sepharose CL-6B (DS) column (40 ml) equilibrated in DS buffer (10 mM K_3_PO_4_, 1 mM benzamidine, and 0.001% Tween 20, pH 6.4). The bound proteins were washed with 200 ml of DS buffer, followed by elution with a linear gradient of 0 to 1.0 M NaCl in DS buffer at 1.5 ml/min for 100 min, and subsequently with 1.0 M NaCl in DS buffer at 1.5 ml/min for 50 min. Collected fractions from the Q column or DS column were subjected to 10% SDS-PAGE and immunoblotting using anti-6×His antibody (GenScript). Fractions containing the target proteins were pooled and adjusted to pH 7.5 before applied to a 2-ml Ni^2+^-NTA agarose column (Qiagen) equilibrated with Ni^2+^-NTA buffer (50 mM tris-HCl, pH 7.5, 300 mM NaCl, 10 mM imidazole, 5% glycerol, and 1 mM benzamidine). After washing with 10 ml of Ni^2+^-NTA buffer, a linear gradient of 10 to 100 mM imidazole in Ni^2+^-NTA buffer was used to elute the bound proteins at a flow rate of 0.5 ml/min for 80 min, followed by 250 mM imidazole in Ni^2+^-NTA buffer at 0.5 ml/min for 20 min. All purification steps were performed at 4°C. After electrophoretic analysis, fractions containing the purified target proteins were combined and concentrated using Amicon Ultra centrifugal filter devices (Millipore). The recombinant proteins were buffer-exchanged to 20 mM tris-HCl, pH 7.5, and 50 mM NaCl, and aliquots were stored at −80°C. The expression of PPO1 and PPO2 was carried out in *E. coli* BL21 Gold (DE3) cells (Strategene) and purified using a similar double Ni^2+^-NTA procedure as described previously (*67*). The small ubiquitin-related modifier (SUMO) fusion partner and the N-terminal 6×His-tag were removed for both PPO1 and PPO2 after expression to expose their authentic N terminus. Nec was obtained from a previous study and expressed in baculovirus-infected Sf9 cells (*56*). No tag was introduced to Nec. The I9 domain of recombinant Pr1A was removed from its precursor by autoproteolysis during expression, as a posttranslational modification. Storage of the purified recombinant Pr1A resulted in the loss of C-terminal 6×His tag, likely due to auto-proteolytic cleavage of Pr1A at the carboxyl side of His residues.

### Immunoblot

The immunoblot data shown in this study are representative results of at least two independent experiments.

### Identification and quantification of target peptides by LC-MS/MS

To determine whether Pr1A and subtilisin are capable of processing after the His residue, gel bands were cut and subjected to in-gel trypsin digestion, as previously described (*68*). Mass spectra of the resulting peptides were obtained by LC-MS/MS. The RAW instrument files were searched against a database consisting of high-quality serine protease sequences (*8*) and RefSeq GCF_000001215.4 (NCBI) using the peptide identification software Byonic (v3.10.4). Chromatographic peaks corresponding to expected peptides cleaved after the His residue were manually examined by querying the instrument RAW data files using Xcalibur Qual Browser (version 2.2.0.23) and the relative abundances of peptides were calculated as previously described (*65*). To determine the band that corresponds to the product cleaved at the canonical site, the proMP1 cleaved by Pr1A-generated Psh was subjected to SDS-PAGE and in-gel Glu-C endopeptidase digestion followed by LC-MS/MS analysis as described above.

### Determination of cleavage sites in Nec and the formation of complexes by mass spectrometry

The N-terminal and C-terminal peptides released in Nec-protease reactions were examined by matrix-assisted laser desorption/ionization-time of flight mass spectrometry (MALDI-TOF) and by LC-MS/MS. The reactions (fig. S13) in buffer A (20 mM tris-HCl, pH 7.5, 20 mM NaCl, 5 mM CaCl_2_, and 0.001% Tween 20) were desalted using C18 solid-phase extraction pipette tip devices (Pierce #87784, Thermo-Fisher) following the manufacturer’s recommendations. For MALDI-TOF analysis, the C18 eluates were mixed with an equal volume of 2,5-dihydroxybenzoic acid MALDI matrix (9 mg/ml in 30% acetonitrile) and subjected to mass determination on a Voyager DE-PRO mass spectrometer in the linear mode (PerkinElmer Life Sciences). The spectra were calibrated using a mixture of standards in a mass range of 300 to 2000 *m*/*z* (mass/charge ratio). The molecular masses of peaks that were absent from the control spectra were compared with values calculated for the N-terminal and C-terminal peptides to determine the cleavage site in Nec. In the duplicate experiment (fig. S14), the C18 eluates were subjected to LC-MS/MS analysis as described above. To examine the constituents of Nec-protease complexes, the reactions (fig. S16) were subjected to 10% SDS-PAGE, followed by in-gel chymotrypsin digestion of the Nec-protease bands and subsequent LC-MS/MS analysis as described above.

### Amidase activity assay

The activation of proSp7 was confirmed by measuring its amidase activity. Briefly, the reactions were mixed with 50 μM acetyl-Ile-Glu-Ala-Arg-*p*-nitroanilide (IEAR*p*Na) (*43*) in 150 μl of reaction buffer (0.1 M tris-HCl, 0.1 M NaCl, and 5 mM CaCl_2_, pH 8.0), and the change in absorbance at 405 nm was recorded every 30 s for 20 min. The reaction rate was calculated from the slope of the initial, linear portion of each curve generated. One unit of activity was defined as a change in OD_405_ of 0.001/min.

### Statistical analysis

The significance of differences in the IEARase activity or expression levels from different groups of samples was calculated with one-way analysis of variance (ANOVA) using SPSS v.27 followed by Tukey’s multiple comparisons test.
