# Supplementary material for: An evolutionarily conserved serine protease network mediates melanization and Toll activation in Drosophila
Source: Sci Adv. 2023 Dec 20;9(51):eadk2756. doi: 10.1126/sciadv.adk2756 (PMC10732536; doi:10.1126/sciadv.adk2756)
Supplement: Supplementary file 1 — Figs. S1 to S17 [file sciadv.adk2756_sm.pdf]

Science Advances
AAAS

Supplementary Materials for

An evolutionarily conserved serine protease network mediates melanization and Toll activation in *Drosophila*

Tisheng Shan *et al.*

Corresponding author: Haobo Jiang, haobo.jiang@okstate.edu

Sci. Adv. **9**, eadk2756 (2023)
DOI: 10.1126/sciadv.adk2756

This PDF file includes:

Figs. S1 to S17

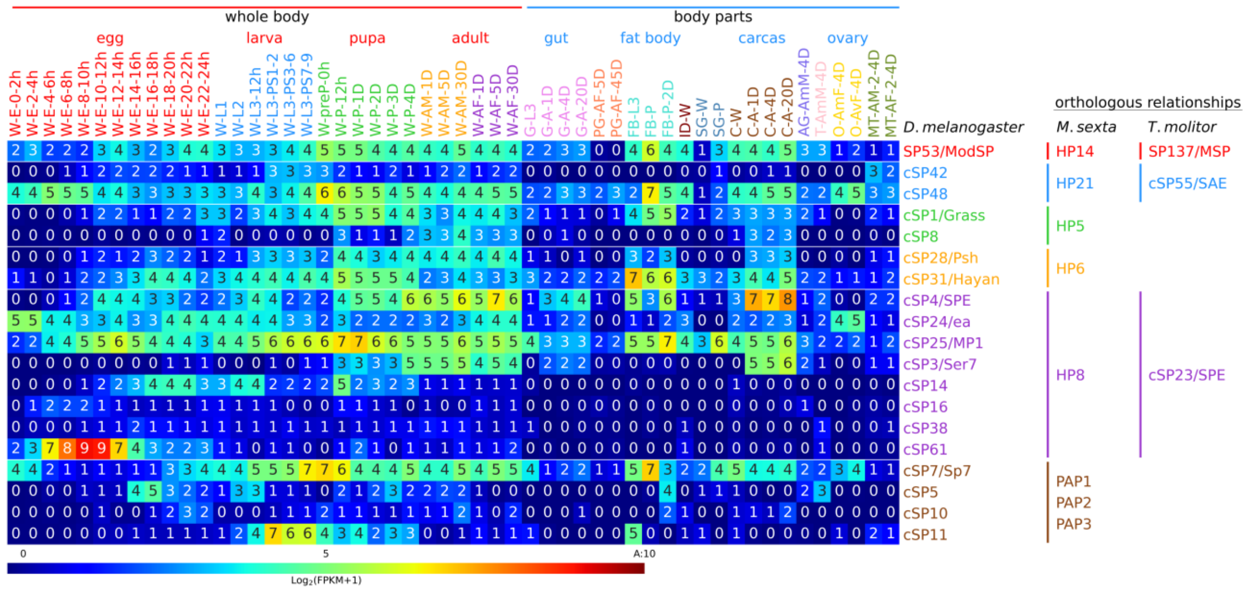

Fig. S1. Transcript profiles and orthologous relationships of putative members of the *Drosophila* immune serine protease system. Candidates of the immune protease cascades in *Drosophila* were selected among the serine protease genes that show a close phylogenetic relationship with the known cascade members in *Manduca* and *Tenebrio*. The phylogenetic and RNA-seq data were retrieved from previous studies (8, 46). The mRNA levels, as represented by $\log_2$(FPKM+1) values, are shown in the gradient heat map from blue (0) to maroon (10). The values are labeled as 0 for 0–0.49, 1 for 0.50–1.49, 2 for 1.50–2.49, 3 for 2.50–3.49, 4 for 3.50–4.49, 5 for 4.50–5.49, 6 for 5.50–6.49, 7 for 6.50–7.49, 8 for 7.50–8.49, and 9 for 8.50–9.49.

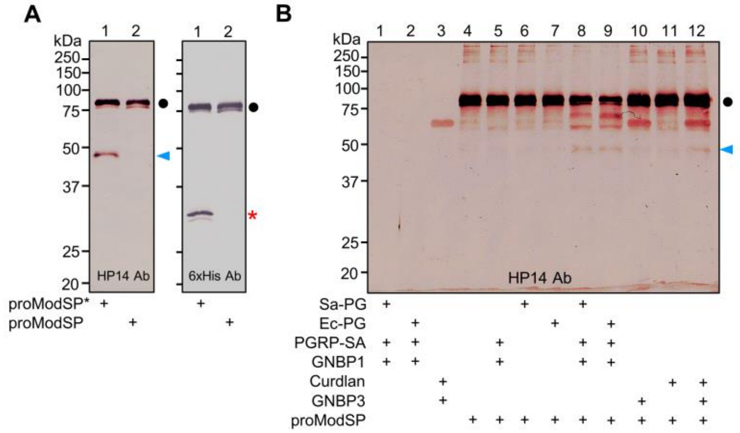

Fig. S2. Autoactivation assay of ModSP zymogen. (**A**) The 10% SDS/PAGE followed by immunoblot analyses of the purified proModSP* and proModSP. ProModSP* (200 ng) or proModSP (200 ng) was treated by SDS sample buffer, separated along with protein size markers, and detected by antibody to *M. sexta* HP14 (left) or anti-6xHis antibody (right). The ModSP zymogen, N-terminal fragment of ModSP, and catalytic domain of ModSP are marked with dots, arrowhead, and asterisk, respectively. The C-terminal catalytic domain (~31 kDa) was hardly detected by anti-HP14 antibody because of its low immunogenicity. (**B**) Purified proModSP (300 ng) was incubated with insoluble *S. aureus* peptidoglycan (500 ng) or soluble *E. coli* peptidoglycan (500 ng), PGRP-SA (500 ng), GNBP1 (500 ng), and buffer A (to 12 μL) for 2 h at 37°C. Alternatively, the zymogen was incubated with curdlan (5 μg), GNBP3 (500 ng), and buffer A (to 12 μL) for 2 h at 37°C. The reaction mixture and controls were subjected to 10% SDS-PAGE under reducing condition, followed by immunoblot analyses using antibody to *M. sexta* HP14. Sizes and positions of the M_r markers are indicated. The ModSP zymogen and the N-terminal fragment of ModSP produced after activation cleavage are marked with dot and arrowhead, respectively.

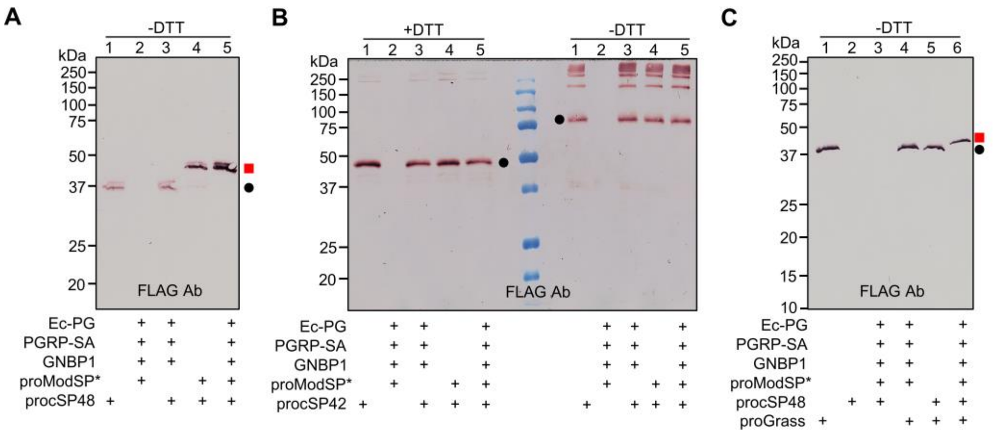

Fig. S3. cSP48 but not cSP42 functions between ModSP and Grass. (**A**) ProcSP48 activation. Purified procSP48 (1 μg) was incubated with *E. coli* peptidoglycan (1 μg), PGRP-SA (500 ng), GNBP1 (500 ng), proModSP* (500 ng), and buffer A (to 25 μL) for 2 h at 37°C. The reaction mixture and controls were separated by 10% SDS-PAGE under non-reducing condition and detected by immunoblotting using anti-FLAG antibody. Sizes and positions of the M_r markers are indicated. The cSP48 precursor and active cSP48 are marked with dot and square, respectively. (**B**) ModSP does not cleave procSP42. Purified procSP42 (1 μg) was incubated with *E. coli* peptidoglycan (1 μg), PGRP-SA (500 ng), GNBP1 (500 ng), proModSP* (500 ng), and buffer A (to 25 μL) for 2 h at 37°C. The reaction mixture and controls were separated by 10% SDS-PAGE under reducing condition (left) or non-reducing condition (right) and detected by immunoblotting using anti-FLAG antibody. Sizes and positions of the M_r markers are indicated. The cSP42 precursor and its dimer are marked with dots. (**C**) ProGrass activation. Purified proGrass (1 μg) was incubated with *E. coli* peptidoglycan (1 μg), PGRP-SA (500 ng), GNBP1 (500 ng), proModSP* (500 ng), procSP48 (100 ng), and buffer A (to 25 μL) for 2 h at 37°C. The reaction mixture and controls were subjected to 12% SDS-PAGE under non-reducing condition and detected by immunoblotting using anti-FLAG antibody. The small amount of procSP48 added in the reaction was hardly detected by the antibody, even though it has a FLAG tag at the C-terminus. Sizes and positions of the M_r markers are indicated. The Grass precursor and active Grass are marked with dot and square, respectively.

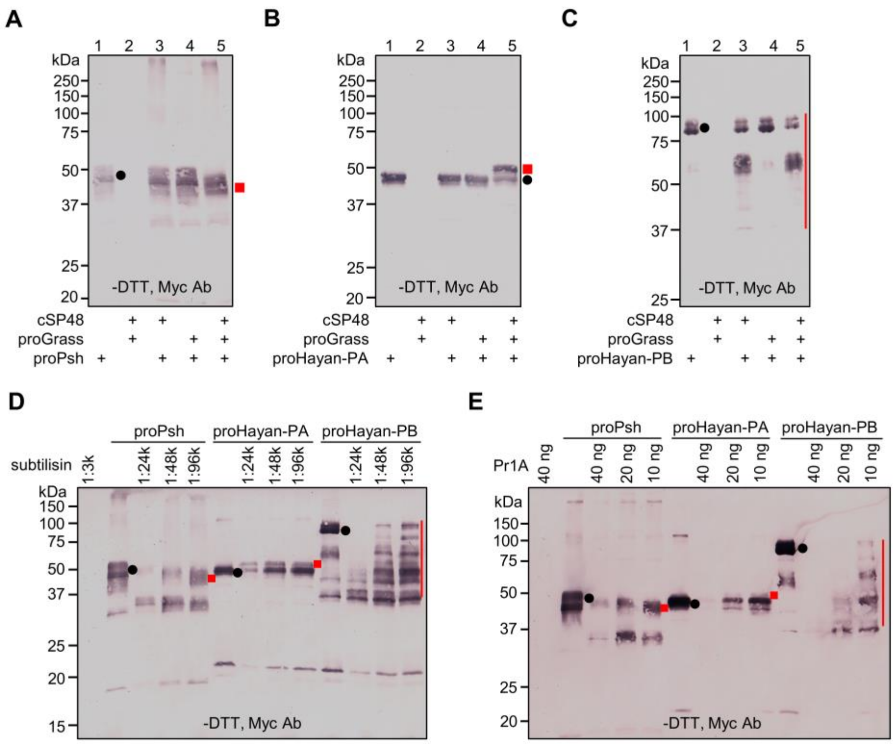

Fig. S4. Psh and two isoforms of Hayan integrate signals from Grass and microbial proteases. (**A–C**) Purified proPsh (400 ng) (**A**) or proHayan-PA (400 ng) (**B**), or proHayan-PB (400 ng) (**C**) was incubated with active cSP48 (100 ng), proGrass (500 ng), and buffer A (to 25 µL) for 1 h at 37°C. Active cSP48 was produced in a mixture of *E. coli* peptidoglycan (1 µg), PGRP-SA (500 ng), GNBP1 (500 ng), proModSP* (500 ng), procSP48 (100 ng), and buffer A (to 20 µL) for 1 h at 37°C. (**D**, **E**) Purified proPsh (500 ng) or proHayan-PA (500 ng) or proHayan-PB (500 ng) was incubated with 1 µL of serially diluted subtilisin purified from *B. subtilis* (**D**) or various amounts of purified recombinant *M. anisopliae* Pr1A (**E**), and buffer A (to 25 µL) for 30 min at 29°C. The reaction mixture and controls were resolved by 10% SDS-PAGE under non-reducing condition and detected by immunoblotting using anti-Myc antibody. Sizes and positions of the M_r markers are indicated. The precursors of Psh, Hayan-PA, and Hayan-PB are marked with dots, while the active forms of Psh and Hayan-PA are marked with squares. The active form of Hayan-PB migrated to a position greater than 37 kDa under non-reducing condition, and it's hard to distinguish that from the surrounding bands (marked with vertical lines).

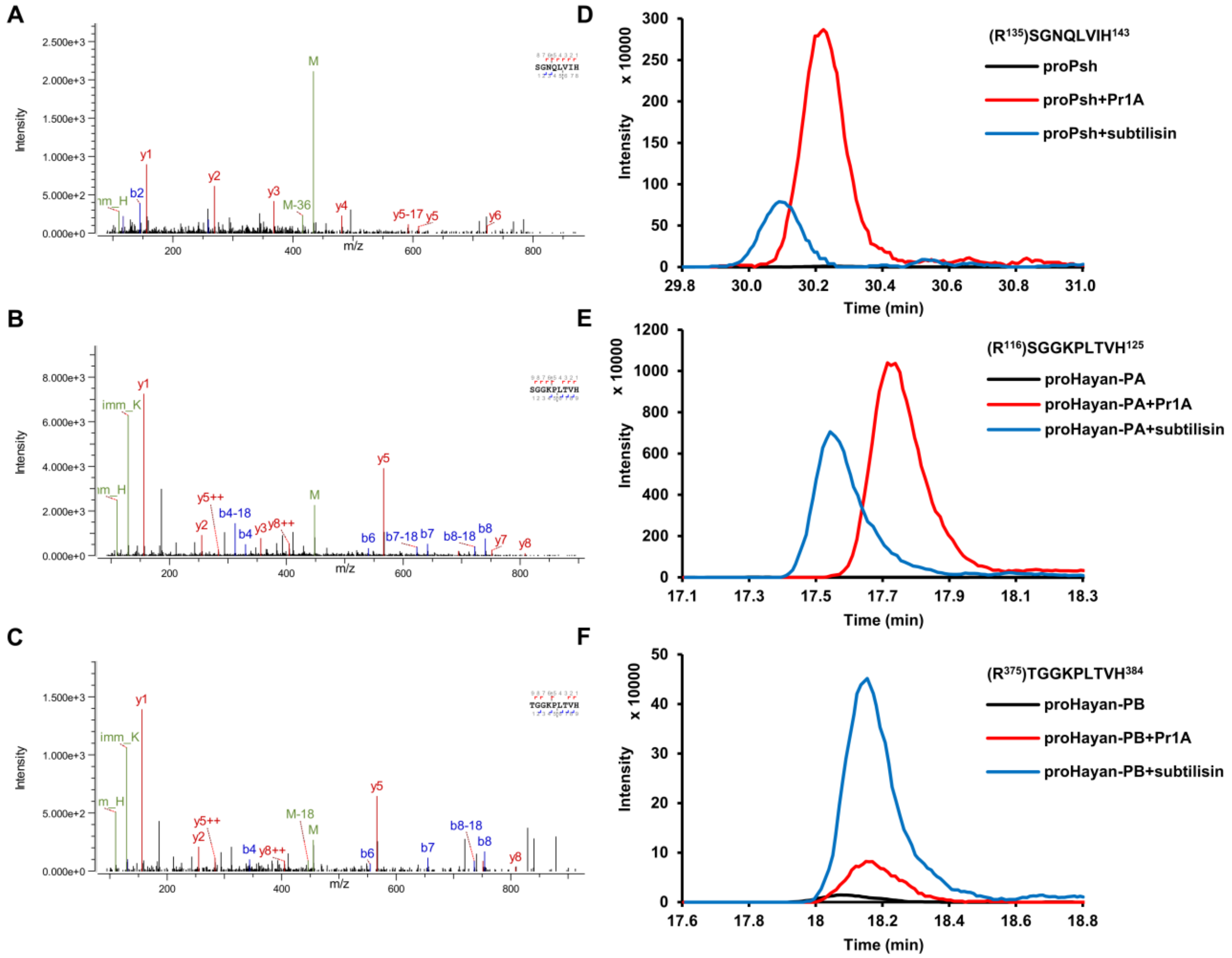

Fig. S5. Identification and quantification of the target peptides after proteolytic maturation. Purified proPsh (2 μg) or proHayan-PA (2 μg) or proHayan-PB (2 μg) was incubated with 20 ng of Pr1A or 1 μL of 1:48,000 diluted subtilisin, and buffer A in a 12 μL reaction volume for 30 min at 29°C. The reaction mixture and zymogen controls were resolved by 12% SDS-PAGE under reducing condition. Following in-gel trypsin digestion and subsequent LC-MS/MS analysis, the second MS spectra (**A–C**) and the intensity (**D–F**) of the peptides cleaved after the His residue were visualized.

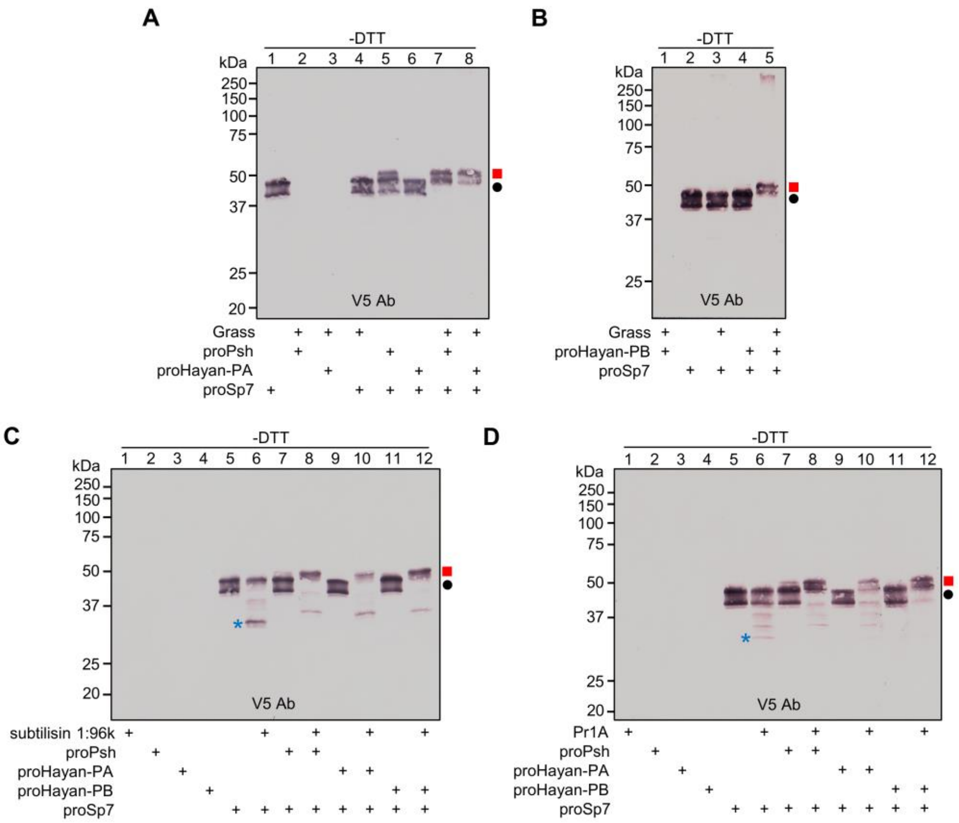

Fig. S6. Cleavage activation of proSp7 by Psh, Hayan-PA, and Hayan-PB. Purified proSp7 (600 ng) was incubated with Grass (100 ng), proPsh (500 ng) or proHayan-PA (500 ng) (**A**) or proHayan-PB (500 ng) (**B**), and buffer A (to 25 μL) for 1 h at 37°C. Active Grass was produced in a mixture of *E. coli* peptidoglycan (1 μg), PGRP-SA (500 ng), GNBP1 (500 ng), proModSP* (300 ng), procSP48 (100 ng), proGrass (100 ng), and buffer A (to 20 μL) for 1 h at 37°C. In two other sets of reactions, purified proSp7 (600 ng) was incubated with 1 μL of 1:96,000 diluted subtilisin (**C**) or 20 ng purified Pr1A (**D**), proPsh (500 ng) or proHayan-PA (500 ng) or proHayan-PB (500 ng), and buffer A (to 25 μL) for 30 min at 37°C. The reaction mixture and controls were subjected to 10% SDS-PAGE under non-reducing condition and detected by immunoblotting using anti-V5 antibody. Sizes and positions of the M_r markers are indicated. The precursor and active form of Sp7 are marked with dots and squares, respectively. The products from proSp7 cleaved by subtilisin or Pr1A are marked with asterisks.

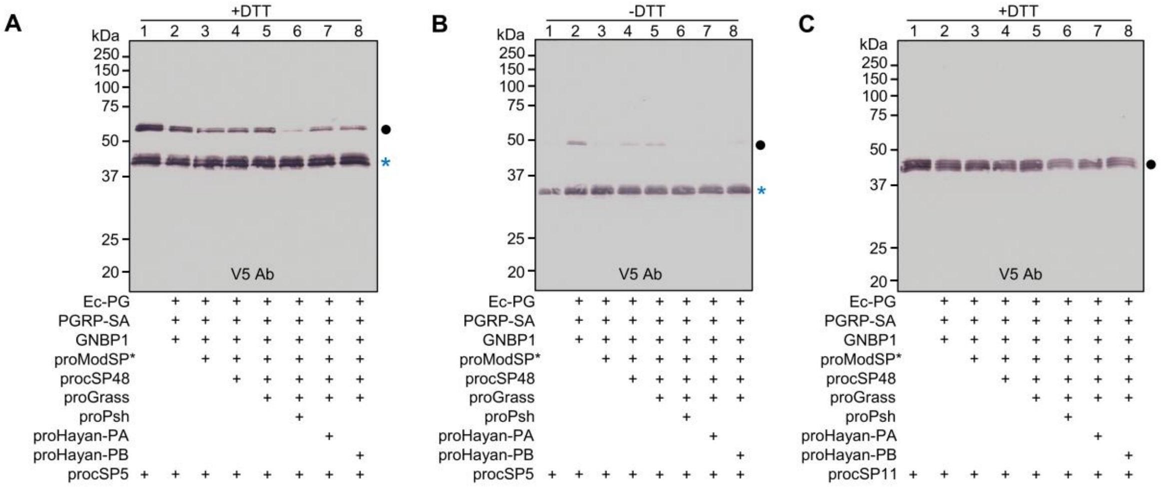

Fig. S7. Detection of procSP5 and procSP11 cleavage by upstream proteases in the pathway. (**A**, **B**) Purified procSP5 (400 ng) was incubated with *E. coli* peptidoglycan (1 μg), PGRP-SA (500 ng), GNBP1 (500 ng), proModSP* (300 ng), procSP48 (100 ng), proGrass (100 ng), proPsh (500 ng) or proHayan-PA (500 ng) or proHayan-PB (500 ng), and buffer A (to 25 μL) for 1 h at 37°C. The reaction mixture and controls were resolved by 10% SDS-PAGE under reducing condition (**A**) or non-reducing condition (**B**) and detected by immunoblotting using anti-V5 antibody. Sizes and positions of the M_r markers are indicated. The cSP5 precursor and processed cSP5 are marked with dots and asterisks, respectively. (**C**) Purified procSP11 (500 ng) was incubated with *E. coli* peptidoglycan (1 μg), PGRP-SA (500 ng), GNBP1 (500 ng), proModSP* (300 ng), procSP48 (100 ng), proGrass (100 ng), proPsh (500 ng) or proHayan-PA (500 ng) or proHayan-PB (500 ng), and buffer A (to 25 μL) for 1 h at 37°C. The reaction mixture and controls were resolved by 10% SDS-PAGE under reducing condition and detected by immunoblotting using anti-V5 antibody. Sizes and positions of the M_r markers are indicated. The cSP11 precursor is marked with dot.

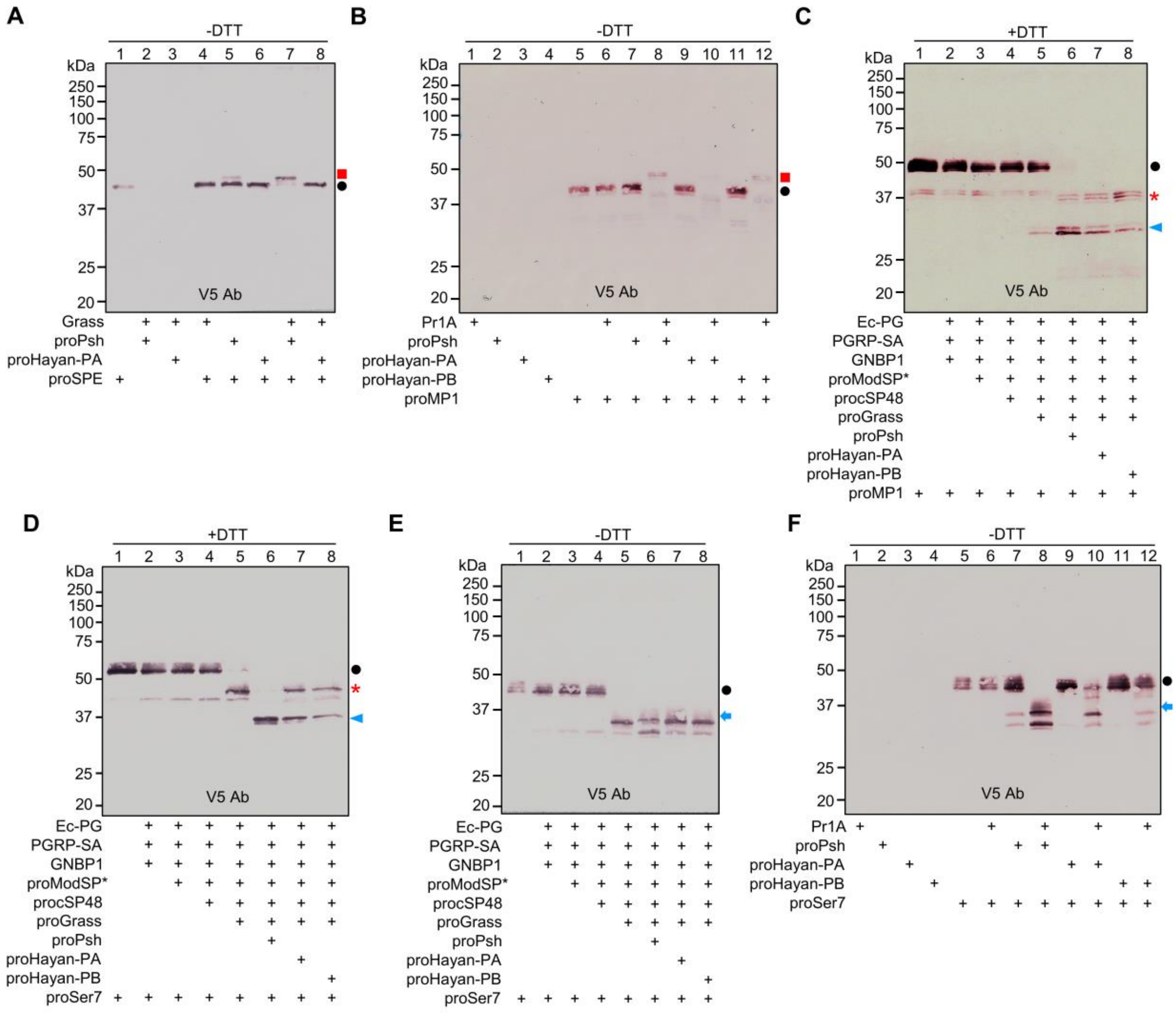

Fig. S8. Cleavage activation of proSPE, proMP1, and proSer7. (**A**) Purified proSPE (600 ng) was incubated with Grass (100 ng), proPsh (500 ng) or proHayan-PA (500 ng), and buffer A (to 25 μL) for 1 h at 37°C. Active Grass was produced in a mixture of *E. coli* peptidoglycan (1 μg), PGRP-SA (500 ng), GNBP1 (500 ng), proModSP* (300 ng), procSP48 (100 ng), proGrass (100 ng), and buffer A (to 20 μL) for 1 h at 37°C. The reaction mixture and controls were subjected to 10% SDS-PAGE under non-reducing condition and detected by immunoblotting using anti-V5 antibody. The SPE precursor and the active SPE are marked with dot and square, respectively. (**B** and **F**) Purified proMP1 (500 ng) (**B**) or proSer7 (500 ng) (**F**) was incubated with *M. anisopliae* Pr1A (20 ng), proPsh (500 ng) or proHayan-PA (500 ng) or proHayan-PB (500 ng), and buffer A (to 25 μL) for 30 min at 37°C. The reaction mixture and controls were subjected to 10% SDS-PAGE under non-reducing condition and detected by immunoblotting using anti-V5 antibody. The precursors of MP1 and Ser7 are marked with dots, while the active MP1 is marked with square. Arrow indicates a cleaved form of Ser7, likely with the catalytic domain and a truncated N-terminal fragment linked by a disulfide bond. (**C**–**E**) Purified proMP1 (500 ng) (**C**) or proSer7 (500 ng) (**D**, **E**) was incubated with *E. coli* peptidoglycan (1 μg), PGRP-SA (500 ng), GNBP1 (500 ng), proModSP* (300 ng), procSP48 (100 ng), proGrass (100 ng), proPsh (500 ng) or proHayan-PA (500 ng) or proHayan-PB (500 ng), and buffer A (to 25 μL) for 1 h at 37°C. The reaction mixture

and controls were separated by 10% SDS-PAGE under reducing condition (**C**, **D**) or non-reducing condition (**E**) and detected by immunoblotting using anti-V5 antibody. The precursors, catalytic domains, and unexpected cleavage products of MP1 and Ser7 are marked with dots, arrowheads, and asterisks, respectively. Arrow indicates a cleaved form of Ser7, likely with the catalytic domain and a truncated N-terminal fragment linked by a disulfide bond. (**A**–**F**) Sizes and positions of the M_r markers are indicated.

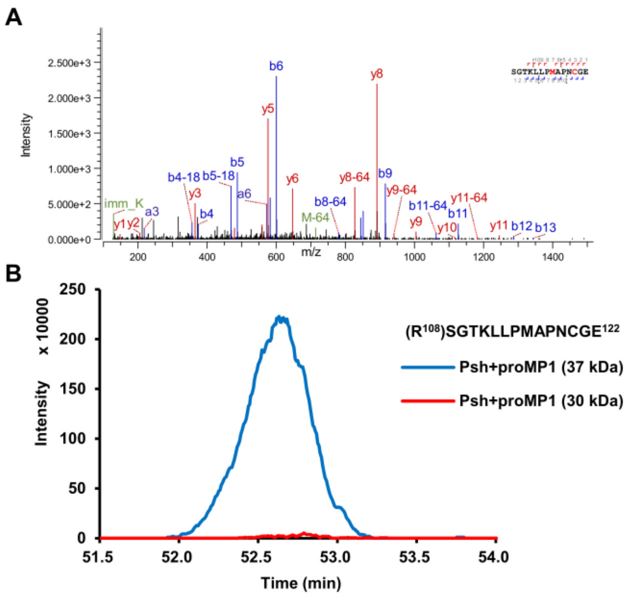

Fig. S9. Identification and quantification of the target peptide after proteolytic maturation. Purified proMP1 (2 μg) was incubated with 40 ng of Pr1A, 2 μg of proPsh, and buffer A in a 12 μL reaction volume for 30 min at 37°C. The reaction mixture and zymogen controls were resolved by 10% SDS-PAGE under reducing condition. Following in-gel Glu-C endopeptidase digestion and subsequent LC-MS/MS analysis, the second MS spectra (**A**) and intensity (**B**) of the target peptide were visualized.

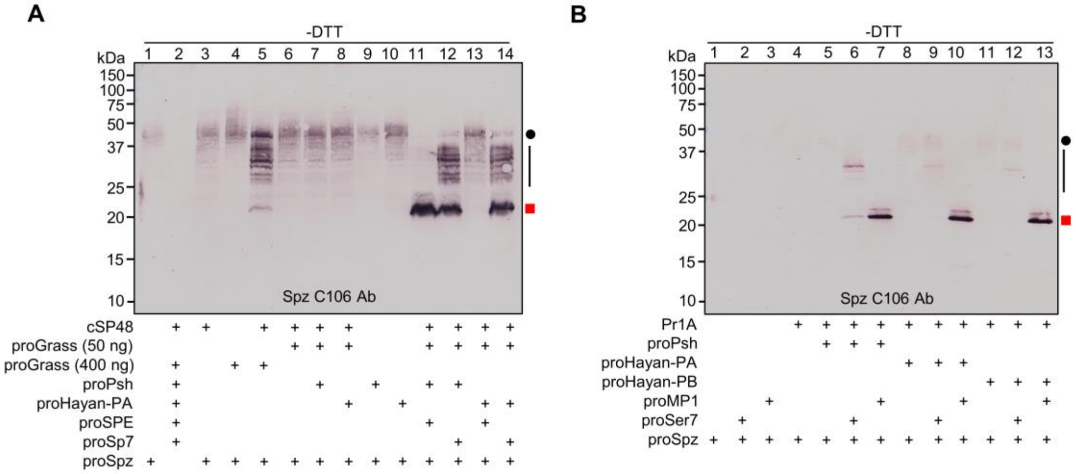

Fig. S10. Cleavage activation of proSpz. (**A**) Purified proSpz (400 ng) was incubated with cSP48 (100 ng), proGrass (50 or 400 ng), proPsh (500 ng) or proHayan-PA (500 ng), proSPE (500 ng) or proSp7 (500 ng), and buffer A (to 25 μL) for 1 h at 37°C. Active cSP48 was produced in a mixture of *E. coli* peptidoglycan (1 μg), PGRP-SA (500 ng), GNBP1 (500 ng), proModSP* (300 ng), procSP48 (100 ng), and buffer A (to 20 μL) for 1 h at 37°C. (**B**) Purified proSpz (400 ng) was incubated with Pr1A (20 ng), proPsh (500 ng) or proHayan-PA (500 ng) or proHayan-PB (500 ng), proMP1 (500 ng) or proSer7 (500 ng), and buffer A (to 25 μL) for 1 h at 37°C. The reaction mixture and controls were subjected to 12% SDS-PAGE under non-reducing condition and detected by immunoblotting using anti-Spz C106 antibody. Sizes and positions of the M_r markers are indicated. The Spz precursor, partially processed Spz, and the disulfide bond-linked cystine-knot domain are marked with dots, vertical lines, and squares, respectively.

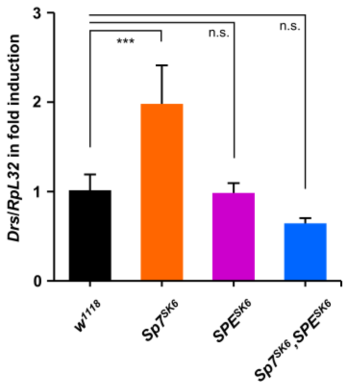

Fig. S11. No significant loss of Toll signaling in double-mutant flies for *Sp7* and *SPE*. Wild-type w^{1118} flies and $Sp7^{SK6}$, SPE^{SK6}, and $Sp7^{SK6}$, SPE^{SK6} mutant flies were immune challenged by septic injury with *B. subtilis* (OD_{600} = 10). Flies were collected 16 h after challenge and *Drs* gene expression was monitored by qRT-PCR in total RNA extracts. *RpL32* mRNA was used as reference gene. Data represent mean ± SD of three independent experiments, each with two technical replicates. Results were normalized to the value in w^{1118} control flies. Statistical significance was calculated with One-way ANOVA with Tukey's multiple comparisons test. ***$p < 0.001$, n.s. $p > 0.05$.

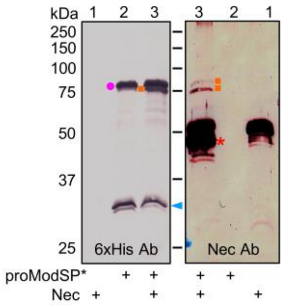

Fig. S12. Formation of covalent complex of Nec and ModSP. This result was obtained by adjusting greyscale of the entire image (Fig. 8A) obtained using the anti-Nec antibody, better revealing the distinct bands at approximately 75 kDa and 80 kDa. The ModSP zymogen, its catalytic domain, ModSP-Nec complex, and cleaved Nec are marked with dot, arrowhead, squares, and asterisk, respectively.

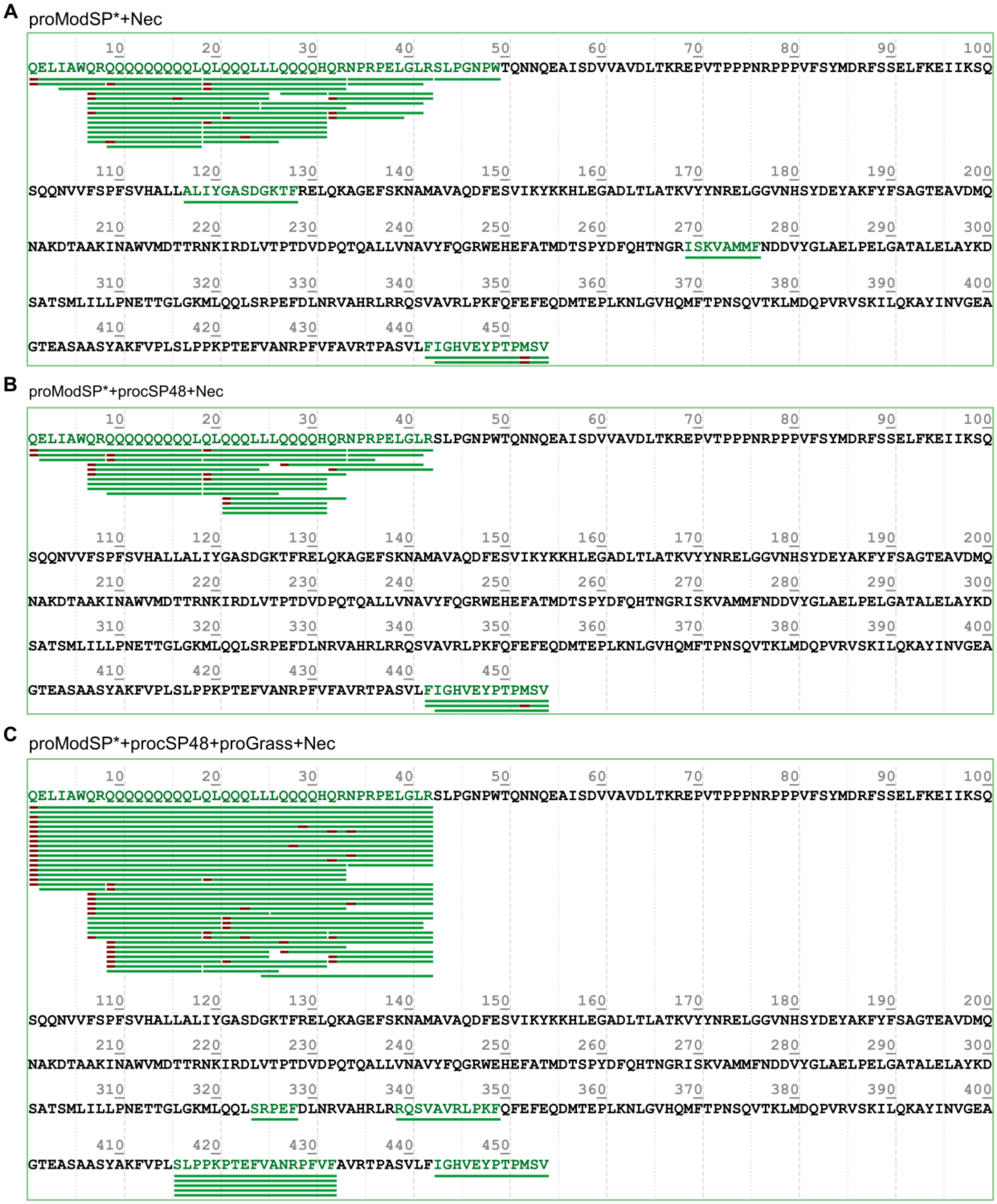

Fig. S13. Peptides coverage map of processed Nec. The N- and C-terminal peptides released in the Nec-protease reactions were directly examined by LC-MS/MS. For this experiment, purified Nec (2 µg) was incubated with proModSP* (2 µg) (**A**), cSP48 (200 ng) (**B**), or Grass (2 µg) (**C**), and buffer A (to 20 µL) for 1 h at 37°C. Active cSP48 was produced by incubating proModSP* (2 µg), procSP48 (200 ng), and buffer A (to 15 µL) for 1 h at 37°C. Active Grass was generated in a

mixture of proModSP* (2 μg), procSP48 (200 ng), proGrass (2 μg), and buffer A (to 15 μL) for 1 h at 37°C. Reactions without Nec and with Nec only (2 μg), were used as controls. No peptides were identified in the control samples.

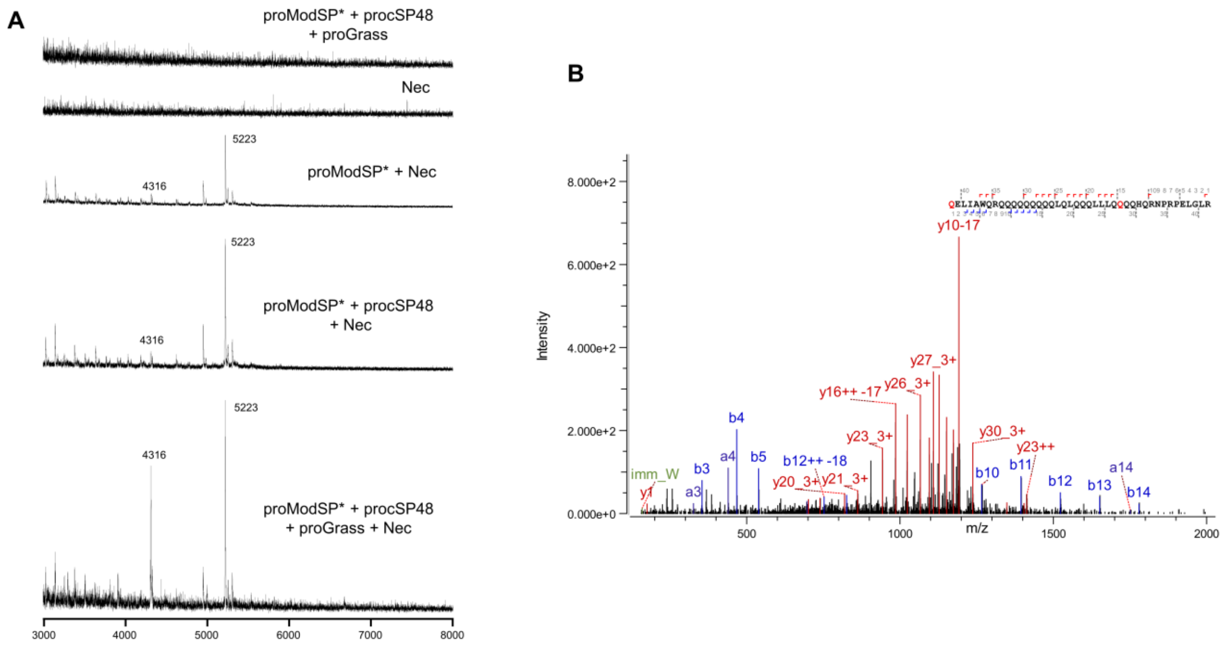

Fig. S14. Mass spectrometric analysis of peptides released from the Nec-protease reactions. (**A**) MALDI-TOF mass spectrometric analysis. The samples were prepared in the same way as described in fig. S13. (**B**) Second MS spectra of the N-terminal peptide of Nec, with an observed M_r of 5,223 Da, directly identified by LC-MS/MS in the Grass and Nec reaction.

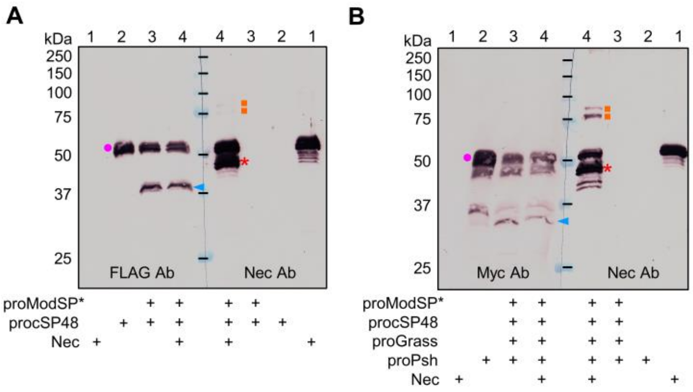

Fig. S15. Nec does not form complexes with cSP48 (A) or Psh (B). (**A**) Active cSP48 was produced in a mixture of proModSP* (500 ng), procSP48 (1 μg), and buffer A (to 23 μL) for 1 h at 37°C. Then Nec (800 ng, 2 μL) was added to the mixture and incubated for 1 h at 37°C. The reaction mixture and controls were subjected to 10% SDS-PAGE under reducing condition and detected by immunoblotting using anti-FLAG (left) or anti-Nec (right) antibodies. (**B**) Active Psh was produced in a mixture of proModSP* (500 ng), procSP48 (100 ng), proGrass (500 ng), proPsh (500 ng), and buffer A (to 23 μL) for 1 h at 37°C. Then, Nec treatment, SDS-PAGE and immunoblot analyses were performed using anti-Myc (left) or anti-Nec (right) antibodies. The cSP48/Psh precursor, its catalytic domain, and cleaved Nec are marked with dot, arrowhead, and asterisk, respectively. The complexes marked with squares were formed between ModSP and Nec, and also between Grass and Nec in panel B. Sizes and positions of the M_r markers are indicated.

A

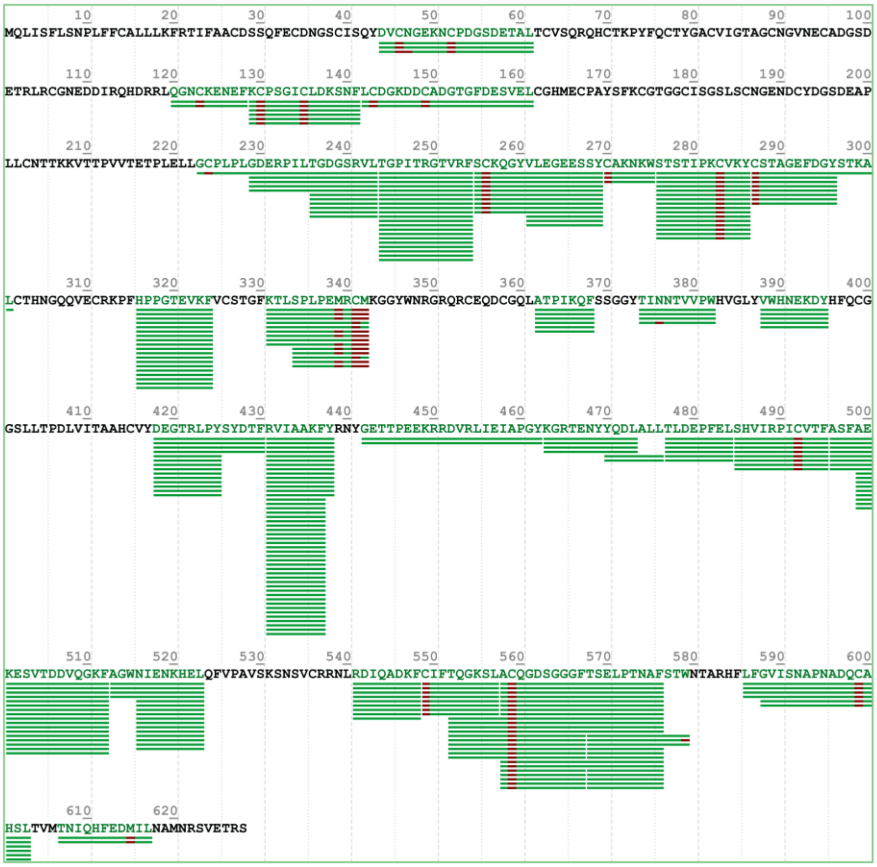

B

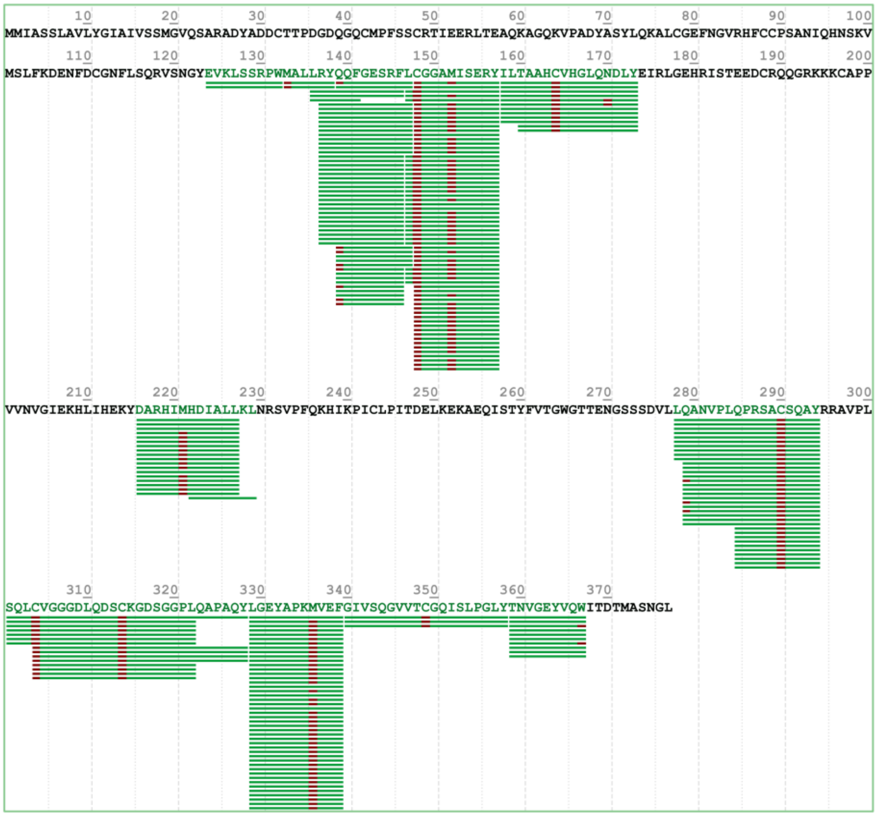

Fig. S16. Peptides coverage map of ModSP and Grass. In the duplicate experiment (fig. S13), the reaction mixture and zymogen controls were resolved by 10% SDS-PAGE under reducing condition. Following in-gel chymotrypsin digestion of the Nec-protease bands and subsequent LC-MS/MS analysis, the peptides coverage map of ModSP (**A**) and Grass (**B**) in the reaction containing proModSP*, procSP48, proGrass, and Nec, were visualized.

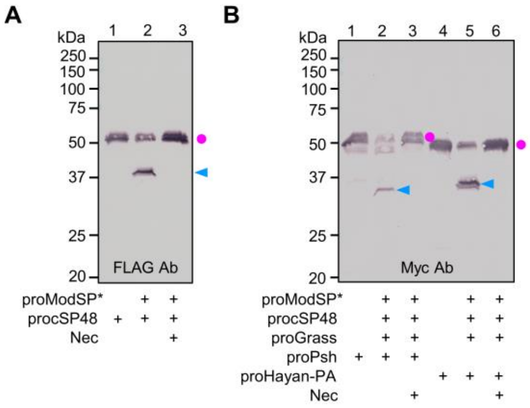

Fig. S17. Inhibitory effect of Nec on the proteolytic activity of ModSP (A) and Grass (B). (**A**) Suppression of procSP48 activation by ModSP. Purified proModSP* (500 ng), procSP48 (500 ng), Nec (800 ng), and buffer A (to 25 μL) were incubated for 2 h at 37°C. The reaction mixture and controls were subjected to 10% SDS-PAGE and immunoblot analyses using anti-FLAG antibody. (**B**) Suppression of proPsh and proHayan activation by Grass. Active Grass was produced in a mixture of proModSP* (500 ng), procSP48 (100 ng), proGrass (800 ng), and buffer A (to 20 μL) for 1 h at 37°C. Then Nec (800 ng, 2 μL) and proPsh (500 ng, 2 μL) or proHayan-PA (500 ng, 1 μL) were added to the mixture and incubated for 1 h at 37°C. The reaction mixture and controls were subjected to reducing SDS-PAGE and immunoblot analyses using anti-Myc antibody. The precursor and catalytic domain of cSP48, Psh and Hayan-PA are marked with dots and arrowheads, respectively. Sizes and positions of the M_r markers are indicated.
